# Research Progress in Traditional Applications, Phytochemistry, Pharmacology, and Safety Evaluation of *Cynomorium songaricum*

**DOI:** 10.3390/molecules29050941

**Published:** 2024-02-21

**Authors:** Jin Zhang, Xingyi Chen, Lu Han, Biao Ma, Mengting Tian, Changcai Bai, Ye Zhang

**Affiliations:** 1 College of Pharmacy, Ningxia Medical University, Yinchuan 750004, China; zhangjin20210911@163.com (J.Z.); cxyfengruoli@163.com (X.C.); lulu2008han@163.com (L.H.); 15008600583@163.com (B.M.); tianmengtjy@163.com (M.T.); 2 Key Laboratory of Ningxia Ethnomedicine Modernization, Ministry of Education, Ningxia Medical University, Yinchuan 750004, China; 3 College of Pharmacy, Inner Mongolia Medical University, Hohhot 010110, China

**Keywords:** *Cynomorium songaricum* Rupr., traditional uses, phytochemistry, pharmacology, toxicology

## Abstract

*Cynomorium songaricum* Rupr. (CSR) belongs to the family *Cynomoriaceae*. It is a perennial succulent parasitic herb with a reddish-brown coloration, predominantly submerged in sand and lacking chlorophyll. Traditionally, it has been used in ethnic medicine to treat various diseases, such as gastric ulcers, indigestion, bowel movements, and improving sexual function. To comprehensively collect CSR data, extensive literature searches were conducted using medical, ecological, and scientific databases such as Google Scholar, PubMed, Science Direct, Web of Science, and China National Knowledge Infrastructure (CNKI). This article summarizes and categorizes research on the uses, phytochemical characteristics, pharmacological activities, and toxicity of ethnic medicine, with the aim of establishing a solid foundation and proposing new avenues for exploring and developing potential applications of CSR. So far, a total of 98 compounds have been isolated and identified from CSR, including flavonoids, terpenes, steroids, and other compounds. It is worth noting that flavonoids and polysaccharides have significant antioxidant and anti-inflammatory properties. In addition, these compounds also show good application prospects in anti-tumor, antioxidant, anti-aging, anti-fatigue, anti-diabetes, and other aspects. Although extensive progress has been made in the basic research of CSR, further research is still needed to enhance the understanding of its mechanism of action and explore more unknown compounds. Our review indicates that CSR has broad prospects and deserves further research.

## 1. Introduction

*Cynomorium* is a genus containing two species, *C. songaricum* Rupr. and *C. coccineum* L., and is in the family *Cynomoriaceae*. These two types are mainly distributed in dry, rocky, or sandy soil areas, mainly appearing in the Northern Hemisphere. For centuries, folk medicine has been widely applied in countries such as Europe, North Africa, East Asia, and West Asia. *Cynomorium songaricum* Rupr. (CSR), a dried succulent stem of a rare endangered medicinal herb that belongs to the genus of *Cynomorium L*. [1], is mainly distributed in Xinjiang, Qinghai, Gansu, Ningxia, Inner Mongolia, Shaanxi and other northwestern regions in China [2]. At present, CSR has been classified as vulnerable (VU) by the International Union for Conservation of Nature (IUCN), and as a Grade II protected plant by the Convention on International Trade in Endangered Species of Wild Fauna and Flora (CITES) [3].

As a plant with homologous medicinal and edible properties, CSR is widely used both domestically and internationally. There are some common ways to consume CSR in North Africa, Europe, East Asia, and West Asia due to its nutritional and health value. For example, fresh CSR can be used for steaming rice, pancakes, etc., while dry CSR can be used for boiling soup, brewing wine, making health foods, etc. [4].

The medicinal history of CSR can be traced back to the Yuan Dynasty, as recorded in the *Ben Cao Yan Yi Bu Yi* (Yuan Dynasty, A.D. 1347) which has the effects of tonifying kidney yang, benefiting essence and blood, and moistening the intestines and relieving constipation [5]. The dried fleshy stem of CSR can tonify kidney Qi, enhance essence blood nourishment, and treat problems such as weakness and impotence [6]. CSR whole grass can treat impotence caused by kidney deficiency, multiple dreams, spermatogenesis, waist and knee weakness, and other symptoms. However, it can also be used in the treatment of diarrhea, women’s leucorrhea, gum bleeding, and other diseases [7].

CSR is rich in polyphenols and polysaccharides, both with biological activities such as scavenging free radicals in the body. As a result, CSR is also known as “the elixir of youth” because it prevents lipid oxidation, aging, cardiovascular disease, cancer, and radiation damage [8]. In recent years, the chemical composition of CSR has been gradually revealed, and it has been reported that it contains flavonoids [9], triterpenoids [10], sugars and glycosides [11], steroids [12], organic acids [13] and other components. Modern pharmacological studies have shown that the extract has the ability to promote cell regeneration and metabolism; enhance immune regulatory function [14]; has anti-cancer [15], anti-viral [16], and anti-aging properties [17]; and relieves fatigue [18].

The present paper provides a comprehensive review of the botanical characteristics, traditional medicinal history, phytochemical composition, pharmacological research, and toxicological research progress of CSR. This systematic analysis aims to offer valuable insights into its clinical application and further research and development in the field of functional foods.

## 2. Materials and Methods

Google Scholar (http://scholar.google.com/, accessed on 27 May 2023), PubMed (http://www.ncbi.nlm.nih.gov/pubmed/, accessed on 27 May 2023), Science Direct (http://www.sciencedirect.com/, accessed on 27 May 2023), Web of Science (http://apps.webofknowledge.com/, accessed on 27 May 2023) and China National Knowledge Infrastructure (https://www.cnki.net/, accessed on 27 May 2023) and other medical, ecological, and scientific databases were used to conduct an extensive literature search to classify the distribution of CSR and preliminary studies. Various keyword combinations such as “*Cynomorium songaricum* Rupr.” and “traditional use”, “phytochemistry”, “pharmacology” and “isolated compounds” were used to collect scientific evidence on plant description, traditional use, phytochemical composition, and pharmacological properties of CSR. EndNote (https://endnote.com/, accessed on 27 May 2023) was used to collate the published literature. And we used the PubChem chemical database (https://pubchem.ncbi.nlm.nih.gov/search/search.cgi/, accessed on 27 May 2023), and other open access and redraw the chemical structure of CSR compounds. The structure of CSR compounds was plotted in Chem Draw 18.0 software.

## 3. Botany

### 3.1. Characteristics of Plants

**Figure 1:**
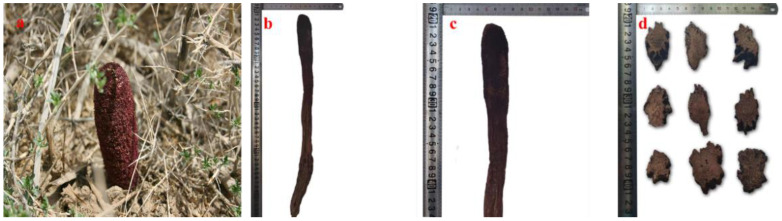
The overall appearance (http://www.iplant.cn/frps/, accessed on 12 May 2023.) (**a**); whole grass (**b**); inflorescence (**c**); and herbal medicinal preparation (**d**) of *Cynomorium songaricum* Rupr.

CSR is a perennial succulent parasitic herb with a reddish-brown coloration, predominantly submerged in sand and lacking chlorophyll (Figure 1). CSR possesses a few triangular scales in its middle and upper sections. Its inflorescence is terminal and clavate, adorned with scaly leaves, featuring bisexual flowers consisting of petals, stamens, and an ovary. Male flowers typically have four perianth pieces, while the pistil undergoes degradation. Female flowers exhibit a lower ovary with usually 5–6 perianth pieces enclosing one pendulous ovule at the apex, male flowers degenerate accordingly. The fruit resembles a nut [19].

### 3.2. Growth Environment and Regional Distribution

**Figure 2:**
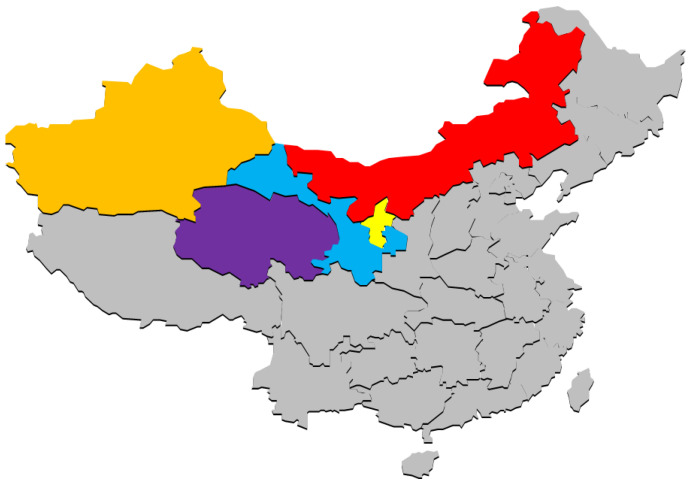
Major producing areas in China of *Cynomorium songaricum* Rupr. Inner Mongolia (Red), Gansu (Blue), Xinjiang (Orange), Qinghai (Purple), and Ningxia (Yellow).

**Figure 3:**
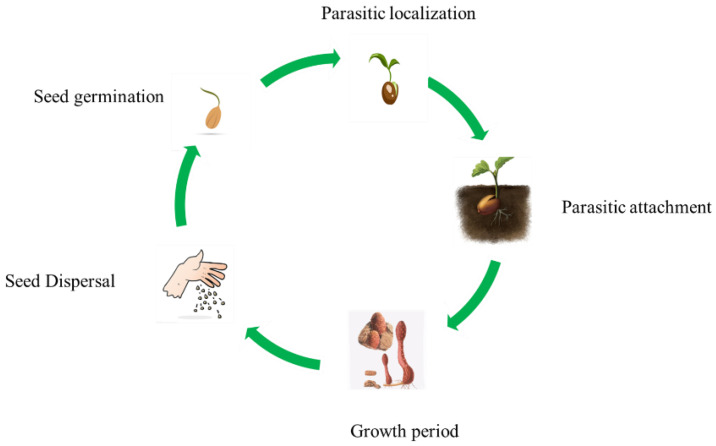
Life cycle of *Cynomorium songaricum* Rupr.

CSR is a rare and endangered medicinal plant mainly distributed in northwest China, such as Inner Mongolia (Red), Gansu (Blue), Xinjiang (Orange), Qinghai (Purple), and Ningxia (Yellow) (Figure 2). Growing in temperate and subtropical regions, it can grow in areas with lower elevations or up to 2000 m above sea level. It likes warm and humid climates, has strong adaptability, and can grow in areas with full or partial sunlight. The minimum temperature is generally required to be no lower than −10 °C. It can withstand high temperatures even in hotter summers. It can adapt to different types of soil but it is best to avoid excessively humid or poor soil. Wild CSR is the main source, with a domestic accumulation of about 30,000 tons. Under normal circumstances, about 1500 tons are harvested annually. However, the existing wild CSR resources are only concentrated in the Hedong sandy land of Pingluo County and their distribution area has been decreasing year by year [20]. There are five host plants, namely *Nitraria sphaerocarpa*, *Nitraria sibirica*, *Nitraria tangutorum*, *Zygophyllum xanthoxylon*, and *Peganum multisectum* [21]. The lifecycle of CSR mainly includes several stages: seed germination, parasitic localization, parasitic attachment, growth cycle, and seed dispersal (Figure 3). Under natural conditions, CSR vegetative growth does not necessitate external light, and the entire growth process takes 4–5 years. However, through artificial cultivation, CSR can be harvested within 3–4 years [22]. CSR seeds germinate under favorable conditions, producing a specialized “bud tube organ”. The terminal regions of this structure expand and adhere to the host plant’s root system, invading it. Once connected to the host plant’s vascular bundle, a parasitic relationship is established, leading to new gemmules [23].

## 4. Traditional Uses

The use of CSR in Asia has a long history, primarily for erectile dysfunction, premature ejaculation, and spermatogenesis enhancement. It was first recorded in *Ben Cao Yan Yi Bu Yi* (Yuan Dynasty, A.D. 1347). Later, the use of the plant was documented in other well-known medicinal works, including *Ben Cao Meng Quan* (Ming Dynasty, A.D. 1565), *Ben Cao Gang Mu* (Ming Dynasty, A.D. 1590), *Ben Cao Qie Yao* (Ming Dynasty, A.D. 1609), *Ben Cao Bei Yao* (Qing Dynasty, A.D. 1694). The traditional preparation of CSR primarily involves the formulation of pills, with notable examples being Huqian wan and Suoyang gujing wan. Its primary function lies in nourishing the kidneys and invigorating Yang, albeit with distinct effects (Table 1).

CSR is primarily utilized in clinical practice for the treatment of andrological and gynecological disorders. Its key therapeutic advantages include enhancing sexual function, regulating endocrine function, and promoting gastrointestinal health. Besides its significant medicinal value, it also finds application in culinary preparations such as steamed rice or pancakes when fresh. It is also incorporated into soups, wines, or health foods when dried. Prolonged consumption can improve immunity and prevent diabetes [24]. Additionally, CSR can serve as a supplementary source of essential trace elements for the human body. CSR exhibits robust vitality and adaptability, thriving in arid deserts, rocky crevices, and harsh environments characterized by drought and wind erosion. It possesses significant ecological value in terms of enhancing environmental conditions, preserving soil stability, and maintaining ecological equilibrium [25].

**Table 1:** The traditional uses of *Cynomorium songaricum* Rupr. in China.

Prescription Name	Main Components	Traditional Uses	Ancient Books	References
Huqian wan	*Cynomorium*, *Cupressus funebris*, *Anemarrhena asphodeloides*, orange peel, *Paeonia lactiflora*, tortoise plastron, etc.	Curing impotence	*Dan Xi Xin Fa* (Ming Dynasty, A.D. 1481)	[26]
Guilu bushen wan	*Cynomorium*, *Epimedium brevicornu*, common jujube seed, *Ipomoea batatas*, *Rubus idaeus*, orange peel, etc.	Curing impotence	*Chinese Pharmacopoeia* 2020	[27]
Suoyang gujing wan	*Cynomorium*, *Cuscuta chinensis*, *Alisma plantago-aquatica*, *Achyranthes bidentata*, *Anemarrhena asphodeloides*, *Poria cocos*, etc.	Curing spermatorrhea	*Chinese Pharmacopoeia* 2020	[28]
Guben wan	*Cynomorium*, *Panax ginseng*, *Oxytropis xinglongshanica*, *Sinocrassula indica*, clam powder, *Atractylodes macrocephala*, etc.	Curing chronic renal failure	*Song Ya Zun Sheng* (Qing Dynasty, A.D. 1695)	[29]
Dabuyin wan	*Cynomorium*, *Cupressus funebris*, *Anemarrhena asphodeloides*, *Paeonia lactiflora*, orange peel, *Stephania tetrandra*, etc.	Curing diabetic nephropathy	*Tong Shou Lu* (Qing Dynasty, A.D. 1762)	[30]
Xusi dan	*Cynomorium*, *Fossilizid*, *Concha ostreae*, *Eucommia ulmoides*, orange peel, *Atractylodes macrocephala*, etc.	Curing male infertility	*Fu Ke Yu Chi* (Qing Dynasty, A.D. 1644–1911)	[31]
Jiawei huqian wan	*Cynomorium*, *Ipomoea batatas*, *Schisandra chinensis*, *Achyranthes bidentata*, *Cupressus funebris*, *Angelica sinensis*, etc.	Strong bones and muscles	*Yi Xue Liu Yao* (Ming Dynasty, A.D. 1609)	[32]
Shenlu jianbu wan	*Cynomorium*, *Cupressus funebris*, *Anemarrhena asphodeloides*, orange peel, *Zingiber officinale*, tortoise plastron, etc.	Strong bones and muscles	*Chinese Pharmacopoeia* 2020	[33]
Jiawei jianbu huqian wan	*Cynomorium*, *Pleuropterus multiflorus*, *Clematis chinensis*, *Cupressus funebris*, *Panax ginseng*, *Hansenia weberbaueriana*, etc.	Curing of fall injury	*Jin Jian* (Qing Dynasty, A.D. 1736)	[34]
Gouqi wan	*Cynomorium*, *Lycium chinense*, *Panax ginseng*, *Cupressus funebris*, *Angelica sinensis*, *Paeonia lactiflora*, etc.	Curing alzheimer disease	*She Sheng Zhong Miao Fang* (Ming Dynasty, A.D. 1550)	[35]
Guilingji capsule	*Cynomorium*, *Talinum paniculatum*, *Lycium chinense*, *Syringa Linn*, *Achyranthes bidentata*, *Cistanche deserticola*, etc.	Curing cognitive dysfunction	*Chinese Pharmacopoeia* 2020	[36]
Jiawei buyin wan	*Cynomorium*, *Achyranthes bidentata*, *Eucommia ulmoides*, *Amomum villosum*, *Angelica sinensis*, *Anemarrhena asphodeloides*, etc	Curing hyperthyroidism	*Zhun Sheng Shang Han* (Ming Dynasty, A.D. 1604)	[37]
Jiajian buyin wan	*Cynomorium*, *Cuscuta chinensis, Angelica sinensis*, *Paeonia lactiflora*, *Eucommia ulmoides*, *Achyranthes bidentata*, etc.	Curing perimenopausal syndrome of Yin deficiency type	*Dan Xi Xin Fa* (Ming Dynasty, A.D. 1481)	[38]

## 5. Phytochemistry

**Figure 4:**
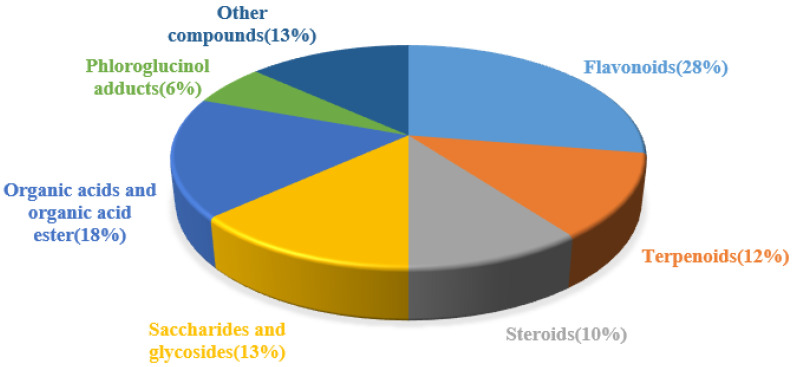
Percentage of chemical composition categories isolated from *Cynomorium songaricum* Rupr.

**Figure 5:**
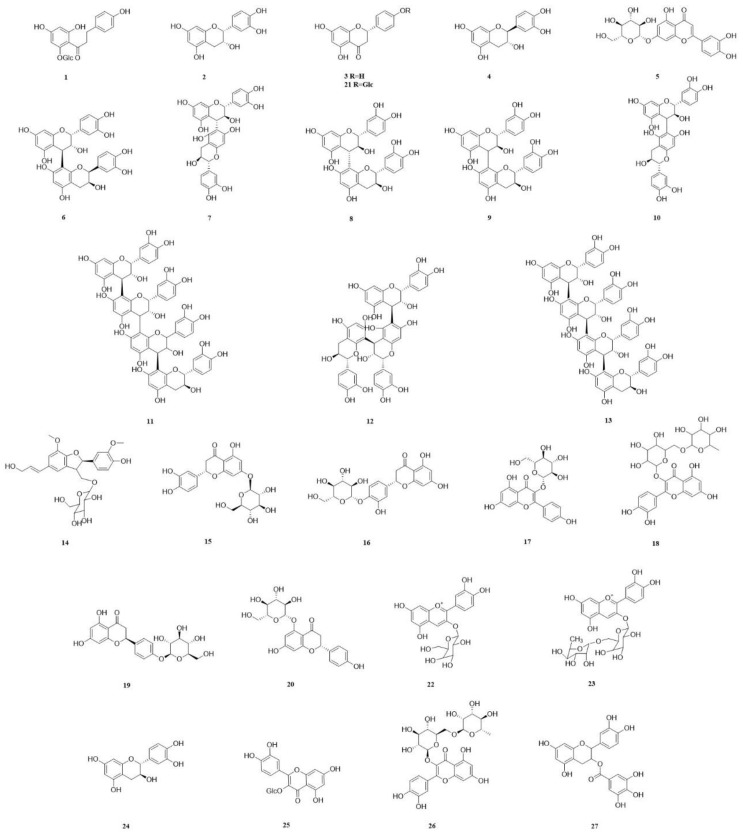
The structures of compounds **1**–**27** from *Cynomorium songaricum* Rupr.

**Figure 6:**
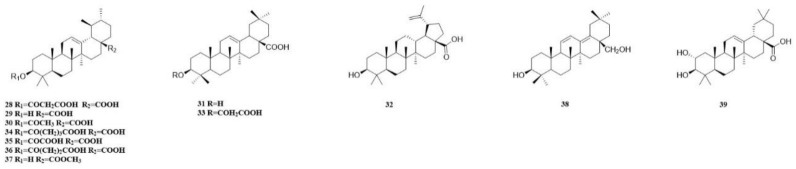
The structures of compounds **28**–**39** from *Cynomorium songaricum* Rupr.

**Figure 7:**
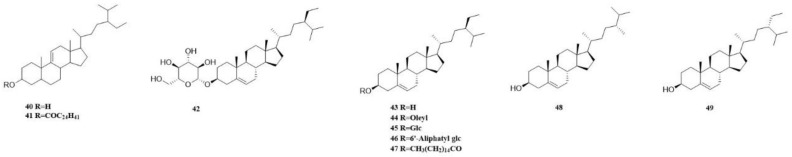
The structures of compounds **40**–**49** from *Cynomorium songaricum* Rupr.

**Figure 8:**
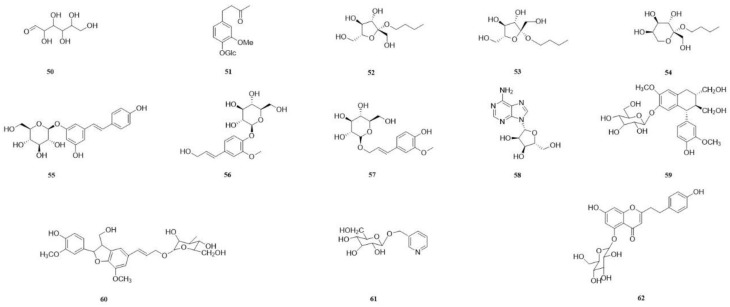
The structures of compounds **50**–**62** from *Cynomorium songaricum* Rupr.

**Figure 9:**
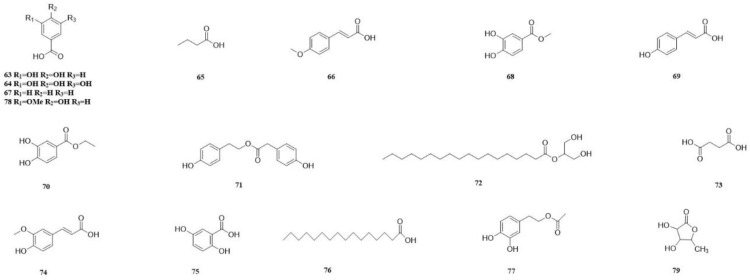
The structures of compounds **63**–**79** from *Cynomorium songaricum* Rupr.

**Figure 10:**
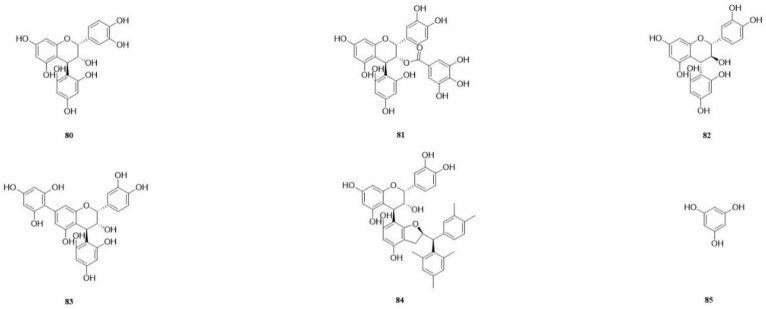
The structures of compounds **80**–**85** from *Cynomorium songaricum* Rupr.

**Figure 11:**
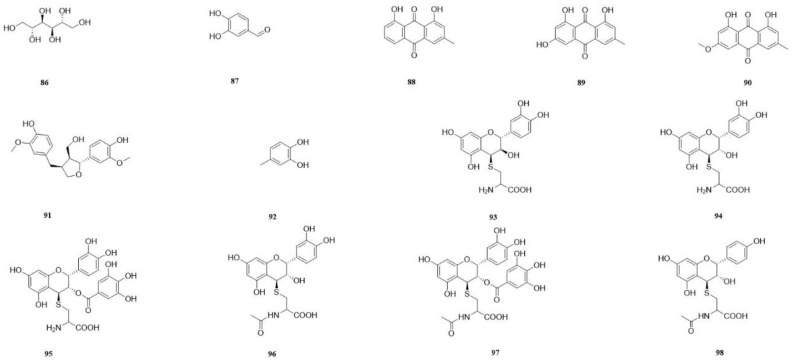
The structures of compounds **86**–**98** from *Cynomorium songaricum* Rupr.

In recent years, a diverse range of potent chemical components have been isolated from various parts of the CSR plant. These components include flavonoids, triterpenoids, steroids, organic acids, sugars and glycosides, amino acids, and trace elements. Most of them have been extensively used in proprietary Chinese medicine and healthcare products. We classify the 98 compounds isolated and identified according to their types. The basic information and source areas of these compounds are summed up in Figure 3, while their structures can be seen in Figure 4, Figure 5, Figure 6, Figure 7, Figure 8, Figure 9, Figure 10 and Figure 11.

### 5.1. Flavonoids

A class of natural compounds with a parent nucleus structure of 2-phenylchromogen (flavone) are called flavonoids, which are the main active ingredients in CSR. Their molecular basis as the antioxidant [39] and anti-aging [40] activities of CSR have been widely proven, and CSR flavonoids have also been found to be effective in antibacterial activity [41].

Currently, a total of 27 types of flavonoids have been isolated from CSR sinensis. Phloridzin (**1**) [42], (−)-epicatechin (**2**), and naringenin (**3**) [9] were obtained from the 70% acetone extract and chloroform extract of stems. Ethyl acetate extract was isolated from (−)-catechin (**4**) [43]. Additionally, luteolin-7-*O*-glucoside (**5**) was isolated from the ethyl acetate fraction of the methanol extract derived from stems of CSR [16]. Two anthocyanins, procyanidin B1 (**6**) and procyanidin B6 (**7**), were extracted from the aqueous extract of stems of CSR chinensis [16].

The procyanidin B3 (**8**) was isolated and identified from the 70% acetone extract of fresh CSR stem through column chromatography. The identified compounds include catechin-(6′-8)-catechin (**9**), catechin-(6′-6)-catechin (**10**), epicatechin-(4*β*-8)-epicatechin-(4*β*-8)-catechin (**11**), epicatechin-(4*β*-6)-epicatechin-(4*β*-8)-catechin (**12**) and arecatannin A1 (**13**) [41]. Furthermore, dehydrodiconiferyl alcohol-9-*O*-*β*-D-glu-copyranoside (**14**), 3′,4′,5,7-tetrahydroxy-flavanone-2(S)-3′-*O*-*β*-D-glucopyranoside (**15**), luteolin-4′-*O*-*β*-glucopyranoside (**16**), astragalin (**17**), quercetin-3-*O*-rutinoside (**18**), naringenin-7-*O*-*β*-D-glucopyranoside (**19**), naringenin-5-*O*-*β*-D-glucopyranoside (**20**) was isolated from the ethyl acetate fraction of 95% ethanol extract obtained from fresh stems of CSR and identified through NMR analysis [4]. The compound naringenin-4′-*O*-*β*-pyranoglucose (**21**) was isolated from the n-butanol fraction of a 95% ethanol extract obtained from CSR whole grass [44].

Two anthocyanin pigments were isolated from a 95% ethanol extract of CSR inflorescences. Cyanidin 3-*O*-glucoside (**22**) was identified as the predominant pigment, accounting for 92%, while cyanidin 3-*O*-rhamnosylglucoside (**23**) was identified as the minor component, comprising 8% [45]. The compounds (+)-catechin (**24**), isoquercetin (**25**), rutin (**26**), and (−)-epicatechin-3-*O*-gallate (**27**) were isolated from ethanol extract of CSR inflorescences [46] (Table 2).

### 5.2. Terpenoids

Terpenoids, composed of isoprene polymers as the basic skeleton, exhibit diverse structures and functions in different plants. These compounds influence plant odor and flavor. Therefore, terpenoids have significant application value in fields such as natural drugs and spices.

The studies have demonstrated that terpenoids are the secondary metabolites of CSR. Twelve terpenoids were isolated, which were as follows. Malonyl ursolic acid hemiester (**28**), ursolic acid (**29**), acetyl ursolic acid (**30**), oleanolic acid (**31**), betulinic acid (**32**) [16], and malonyl oleanolic acid hemiester (**33**) [10] were isolated from dichloromethane extract of CSR stem. The glutaryl ursolic acid hemiester (**34**), oxalyl ursolic acid hemiester (**35**), succinyl ursolic acid hemiester (**36**), and ursolic acid methyl ester (**37**) were isolated from the ethyl acetate extract of CSR stem [47]. Additionally, a diterpenoid compound 3*β*, 28-dihydroxyoleana-11,13(18)-diene (**38**) was isolated from the ethyl acetate fraction of a 95% ethanol extract obtained from the CSR stem [48]. The isolation of maslinic acid (**39**) was achieved from the aqueous extract of CSR [49].

The terpenoids discovered in CSR malonyl ursolic acid hemiester, ursolic acid, acetyl ursolic acid, and malonyl oleanolic acid hemiester are commonly occurring triterpenes that can also be found in other plant species (Table 3).

### 5.3. Steroids

The tetracyclic structure of cycloalkyl polyhydrophenanthrene is the parent nucleus of this class of compounds known as steroids. There are several substances found in fauna and flora, including cholesterol, steroid hormones (such as estrogen, androgen, and adrenal corticosteroids), and sterols. These substances play a crucial role in physiological functions with a wide range of biological functions.

Ten steroid compounds were isolated from CSR was achieved. The compounds 5*α*-Stigmast-9(11)-en-3*β*-ol (**40**) and 5*α*-Stigmast-9(11)-en-3*β*-ol tetracosatrienoic acid ester (**41**) were isolated from ethyl acetate extract of stems of CSR [12]. Daucosterol (**42**) and *β*-sitosterol (**43**) were isolated from the ethyl acetate fraction of a 95% ethanol extract obtained stem of CSR [13]. The *β*-sitosteryl oleate (**44**), *β*-sitosteryl glucoside (**45**), and *β*-sitosteryl glucoside 6′-*O*-aliphatates (**46**) were isolated from the dichloromethane extract of CSR stem [16]. Furthermore, *β*-sitosterol palmaitate (**47**) was isolated from the chloroform extract [50]. The identification and analysis of campesterol (**48**) and *γ*-sitosterol (**49**) were conducted using Gas chromatography–mass spectrometry (GC-MS) in addition to other techniques [51] (Table 4).

### 5.4. Saccharides and Glycosides

Saccharides are one kind of important bioactive compounds in CSR, which exhibit diverse biological and pharmacological activities. These two polysaccharides consist of galactose, glucose, arabinose, rhamnose, mannose, ribose, and uronic acid. Among these components, the latter two ingredients account for 10.7% and 10.5%, respectively [52]. By report, a water-soluble heteropolysaccharide called CSPA, which is a heteropolysaccharide composed of arabinose (Ara), glucose (Glu), and galactose (Gal), was isolated from CSR. It has a molecular weight of 1.394 × 10^5^ Da. The chemical structure consisted of the following units: “→3)-*α*-araf-(1→3)-*α*-d-glcp-(1→4)-*α*-d-GalpA6Me-(1→” [53]. The polysaccharide extracted from CSR was fractionated into three components, namely CSG-F1, CSG-F2, and CSG-F3. CSG-F1 (yield of 21%) exhibited an average molecular weight of approximately 2.4 × 10^5^ Da and primarily consisted of galactose, glucose, arabinose, and rhamnose. Similarly, the purified CSG-F2 (yield of 14%) displayed an average molecular weight of around 1.3 × 10^5^ Da and contained galactose, glucose, arabinose, rhamnose, and ribose as its main constituents. Lastly, the purified CSG-F3 (yield of 37%) had an estimated average molecular weight of about 1.9 × 10^5^ Da and glucose, arabinose, rhamnose, ribose, and mannose [54].

The isolation of thirteen sugars and glycosides from CSR was achieved. Glucose (**50**) was isolated from the chloroform extract of CSR stem [9]. Zingerone 4-*O*-*β*-D-glucopyranosid (**51**) [10] was isolated from the dichloromethane extract of CSR stem. Three fructosides were isolated from the ethyl acetate extract. The structures of *n*-butyl-*β*-D-fructofuranoside (**52**) [55], *n*-butyl-*α*-D-fructofuranoside (**53**) [11], and *n*-butyl-*β*-D-fructopyranoside (**54**) [56] were determined using spectroscopic methods. The compound piceid (**55**) [16] was obtained from the ethyl acetate fraction of the methanol extract, while coniferin (**56**) and isoconiferin (**57**) [16] were isolated from the n-butanol fraction. The isolation of adenosine (**58**) [16] was achieved from the n-butanol fraction of the CSR methanol extract. The compounds (−)-isolariciresinol 4-*O*-*β*-D-glucopyranoside (**59**) and (7S,8R)-dehydrodiconiferyl alcohol 9′-*β*-glucopyranoside (**60**) were isolated from the aqueous extract [42]. The compound nicoloside (**61**) [42] was isolated from the aqueous fraction of the methanol extract obtained from CSR. Furthermore, songaricumone A (**62**) was isolated from the ethyl acetate fraction of 95% ethanol extract obtained from fresh stems of CSR and identified through NMR analysis [4] (Table 5).

### 5.5. Organic Acids and Organic Acid Ester

Organic acids and esters can serve as carriers for drug delivery systems, improving the physical stability and solubility of drugs, regulating lipid metabolism, participating, and regulating various physiological processes as signaling molecules, and having anti-inflammatory and antibacterial effects. 

One of the important active ingredients of CSR is an acidic organic compound called organic acid. At present, 17 distinct types of organic acids and organic acid ester have been successfully isolated from CSR. Protocatechuic acid (**63**), gallic acid (**64**), *n*-butyric acid (**65**) [13], and 4-methoxycinnamic acid (**66**) [48] were isolated from the ethyl acetate fraction of a 95% ethanol extract obtained from stems of CSR. The compounds *p*-hydroxybenzoic acid (**67**) [42], methyl protocatechuicate (**68**) [42] and *p*-hydroxybenzoic acid (**69**) [16], were isolated from the ethyl acetate fraction of the methanol extract. The compounds 3,4-dihydroxybenzoic acid ethyl ester (**70**) [57], 4-hydroxyphenethyl 2-(4-hydroxyphenyl) acetate (**71**) [48], and stearic acid *α*-monoglyceride (**72**) [13] were isolated from the ethyl acetate fraction of a 95% ethanol extract obtained from stems of CSR. The water parts are separated by succinic acid (**73**) [43]. The compounds ferulic acid (**74**) [58] were isolated from a 70% ethanol extract of stems of CSR. Additionally, gentisic acid (**75**), palmitic acid (**76**), and 3,4-dihydroxyphenethyl acetate (**77**) were obtained from a water extract [49]. The vanillic acid (**78**) [44] was extracted from an aqueous solution of 95% ethanol extract from whole grass, while the capilliplactone (**79**) [59] was isolated from the ethyl acetate fraction. The structure was determined using spectroscopic techniques (Table 6).

### 5.6. Phloroglucinol Adducts

A type of compound formed by three hydroxyl groups (-OH) to replace the hydrogens at the 1,3,5 positions in benzene, is called phloroglucinol. It is a drug widely used in the medical field. Belonging to the class of phenobarbital drugs, it has pharmacological effects such as sedation, hypnosis, anticonvulsant, and antianxiety. It is considered an important drug that can be used to treat various diseases and symptoms.

Six types of phloroglucinol compounds were isolated from CSR. The identification of three phloroglucinol compounds was achieved from the 70% acetone extract of fresh stems of CSR using LC-MS and HPLC retention time analysis. These compounds were identified as epicatechin-(4*β*-2)-phloroglucinol (**80**), epicatechin-3-*O*-gallate-(4*β*-2)-phloroglucinol (**81**) and catechin-(4*α*-2)-phloroglucinol (**82**) [41]. Two new compounds were recently isolated from a degraded mixture of cynomoriitannin and identified as cynomoriitannin-phloroglucinol A (**83**) and cynomoriitannin-phloroglucinol B (**84**) based on spectroscopic analyses [41]. Phloroglucinol (**85**) was isolated from the stem’s aqueous extract of CSR [49] (Table 7).

### 5.7. Other Compounds

The qualitative and quantitative analysis of amino acids revealed the presence of 20 common amino acids in CSR, which serve as essential nutritional elements for the human body [60]. Furthermore, volatile components [61], trace elements [62], and tannins [63] play a significant role in the pharmacological activity of CSR.

Mannitol (**86**) was isolated from the aqueous extract [49]. Protocatechualdehyde (**87**), chrysophanol (**88**), emodin (**89**), and physcion (**90**) were isolated from the 70% ethanol extract [58]. The compounds (−)-lariciresinol (**91**) and 4-methylcatechol (**92**) [57] were isolated from the ethyl acetate fraction of a 95% ethanol extract obtained from the CSR stem. Additionally, the following compounds were also identified: 4*β*-(L-cysteinyl)-catechin (**93**), 4*β*-(L-cysteinyl)-epicatechin (**94**), 4*β*-(L-cysteinyl)-epicatechin 3-*O*-gallate (**95**) three cysteine conjugates [64], 4*β*-(L-acetylcysteinyl)-epicatechin (**96**), 4*β*-(L-acetylcysteinyl)-epicatechin 3-*O*-gallate (**97**), 4*β*-(L-acetylcysteinyl)-epiafzelechin (**98**) three acetylcysteine conjugates [64], and edible reagents from CSR were isolated and purified. The structures were elucidated via a combination of NMR and mass spectrometry techniques [64] (Table 8).

## 6. Pharmacology

**Figure 12:**
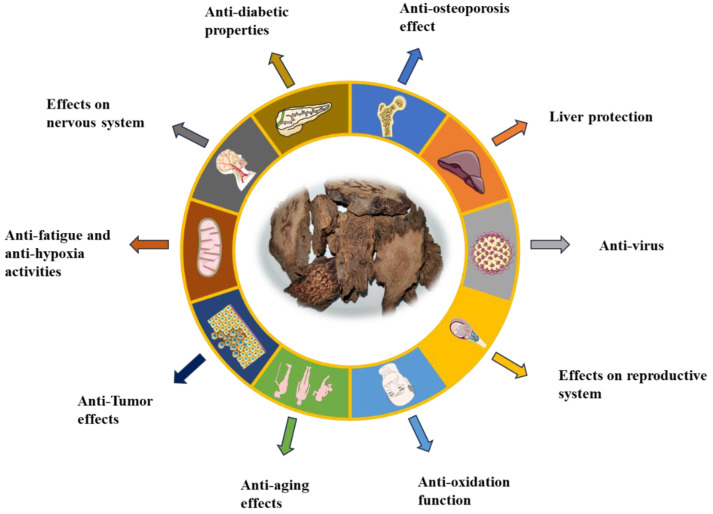
The main pharmacological action of *Cynomorium songaricum* Rupr.

**Figure 13:**
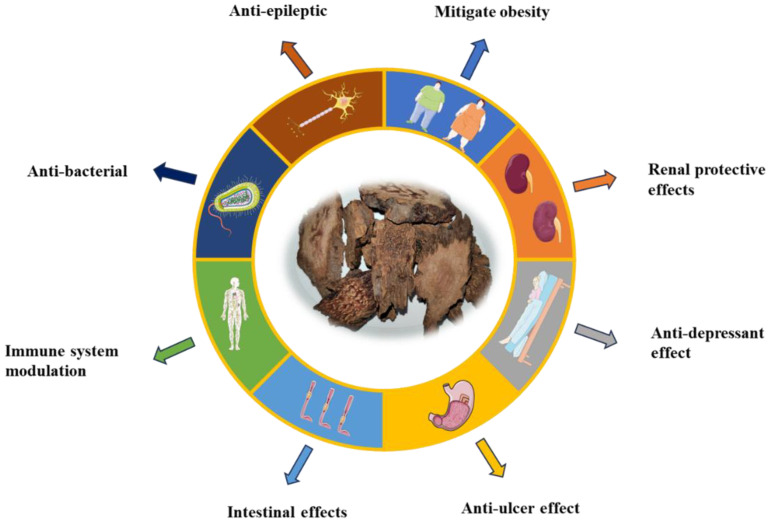
Other pharmacological action of *Cynomorium songaricum* Rupr.

Scholars have combined traditional Chinese medicine with modern medicinal chemistry to explore the biological activity of chemical components in traditional Chinese medicine. Alcohol and water extracts exhibit significant pharmacological activities, including anti-oxidant and anti-tumor effects, among others (Figure 12 and Figure 13).

### 6.1. Anti-Tumor Effects

Inducing apoptosis serves as a method for preventing and treating tumors as it plays an essential role in tumor progression [65]. Cancer stem cells are inhibited from proliferating and dying when exposed to CSR. It can be used to treat malignant tumors such as breast cancer, leukemia, colon cancer, and others.

#### 6.1.1. Anti-Cancer

There are four breast cancer cell lines inhibited by CSR extracts and its ethyl acetate extraction site, including MDA-MB-231 [15,66], MCF-7 [15,66], MB468 [15], and 4T1 [15]. Furthermore, CSR extract induces Foxo3 expression in apoptosis and prevents the transition from G1 to S phases [15]. It has been found that chloroform and ethyl acetate extraction sites from the CSR ethanol extract are capable of inhibiting the proliferation of the colon adenocarcinoma cell line Caco-2 [7]. In cell research for cervical cancer treatment, *Cynomrium* songaricum polysaccharides (CSP) inhibit proliferative activity in HeLa cells [67]. A further study showed that both methanol extract and anthocyanin 3-*O*-glucoside from CSR inhibited KBWT cell proliferation in a dose-dependent manner [68]. By inhibiting telomerase reverse transcriptase (TERT) mRNA, CSP induced apoptosis in A549 cells [69]. Methanol extract and aqueous extract from CSR inhibited the growth of B16 cells, which are used for studying skin cancer in humans [15]. Further, CSR ethyl acetate extract inhibited both LNCaP and HepG2 cells, showing that it may have therapeutic effects on prostate cancer and liver cancer [66]. Research has shown that the anticancer ingredients in CSR are concentrated in the ethyl acetate extraction site, and it may be related to activating and enhancing autophagy processes in cells to trigger. In autophagy and apoptotic cell death, mitochondrial-related proteins Bcl-2/adenovirus E1B 19 kDa-interacting protein 3 (BNIP3) and Bcl-2/adenovirus E1B 19 kDa protein-interacting protein 3-like (BNIP3L) play a critical role [66].

#### 6.1.2. Leukemia

CCRF-CEM and CCRF-SB cells were inhibited by methanol extract and anthocyanin 3-*O*-glucoside from CSR [68]. Similarly, mitochondrial pathways modulate caspase-3 activity. Therefore, CSR ethanol extract causes apoptosis in leukemia cells by causing apoptosis in HL-60 cells [70].

The above studies indicate that CSR has a certain inhibitory effect on two types of tumor cells, cancer, and leukemia. In cancer, it inhibits the growth of cancer cells by inducing the expression of Foxo3 and inhibiting telomerase reverse transcriptase mRNA to activate mitochondrial-related proteins BNIP3 and BNIP3L. Regulating caspase-3 activity through the mitochondrial pathway in leukemia induces cell apoptosis. In contrast, there is more research data on adenocarcinoma and less research on leukemia. However, it cannot be concluded with certainty that CSR has a better therapeutic effect on cancer than on leukemia. Therefore, more in-depth research is still needed (Table S1, Supplementary Materials).

### 6.2. Anti-Oxidation Function

Different parts of CSR have different antioxidant activities when extracted from methanol. 2,2-Diphenyl-1-picrylhydrazyl (DPPH) radicals are best scavenged in the central part, hydroxyl radicals are best inhibited in the lower part, and superoxide anions are highly resisted in the upper part [71]. Multiple solvent extracts of CSR exhibit antioxidant activity. A methanol extract of the CSR and an ethyl acetate extraction site strongly inhibit superoxide anions [72,73]. DPPH radicals, 2,2′-Azino-bis (3-ethylbenzothiazoline-6-sulfonic acid) diammonium salt (ABTS) radicals, and hydroxyl radicals are also scavenged by the aqueous extract and ethyl acetate extraction site [4]. Additionally, the aqueous extract was able to scavenge DPPH free radicals and inhibit superoxide anion formation [74].

Different extracts exhibit significantly different antioxidant and radical scavenging properties. CSR aqueous extract scavenges DPPH free radicals and nitrates more effectively than ethanol extract. In contrast, ethanol extract inhibits xanthine oxidase (XO) and superoxide dismutase (SOD) more effectively than aqueous extract [75].

To examine the categories of substances with superior antioxidant effects, compounds of the same type extracted from CSR were compared to their antioxidant activity. This was performed to clarify the strong antioxidant activity of CSR extract. Within a certain concentration range, CSP exhibits effective scavenging ability against superoxide anion radicals, DPPH radicals, and hydroxyl radicals [6]. CSR flavonoids also scavenge DPPH and hydroxyl radicals [76].

Crude polyphenols exhibited significantly higher antioxidant activity than crude polysaccharides when measured against DPPH radicals, ABTS free radicals, and crude polysaccharides in CSR [77]. According to another study, microwave-extracted procyanidins exhibited superior scavenging activity against DPPH and hydroxyl free radicals [39].

The main antioxidant component in the CSR ethyl acetate extraction site is catechin, which was isolated from protocatechuic acid, gallic acid, and catechins [78]. The aqueous extract of CSR was separated into catechin, epicatechin, and olive saponin to determine the DPPH free radical scavenging capacity [79].

In vivo experiments were used to verify the CSR extract’s significant antioxidant activity. CSR extracts (0.22 g/kg, 0.44 g/kg, 0.88 g/kg) can enhance the serum DPPH free radical scavenging ability of KM mice, and reduce oxidative damage caused by free radicals and lipid peroxides [80].

Some in vivo and in vitro experiments have shown that the antioxidant components in CSR exert antioxidant effects by clearing free radicals, as well as inhibiting XO and SOD, etc. The antioxidant activity of CSR is one of its main functions, which can slow down the oxidative state of the body and fight against diseases (Table S2, Supplementary Materials).

### 6.3. Anti-Aging Effects

Several studies have demonstrated the anti-aging effects of CSR through a variety of mechanisms. According to reports, adding CSR to the diet can extend the average and maximum lifespans of adult female flies. Ethanol extract of CSR suppresses age-related learning disabilities in elderly flies by reducing hydrogen peroxide levels and increasing antioxidants, extending their lifespan, improving mating readiness, increasing fertility, and inhibiting age-related learning disabilities [81]. A transcriptome sequencing study found that CSR extract impacted wild-type Caenorhabditis elegans aging. The lifespan of Caenorhabditis elegans was extended and motor abilities were enhanced by ethyl acetate extract (0.4 mg/mL). Multiple pathways and genes collaborate to produce the effects of the ethyl acetate extract [82]. Various research results have shown that extracts, CSP, and preparations from CSR can delay aging by inhibiting telomere length shortening [83], enhancing telomerase activity [84], improving immune function [85,86,87], inhibiting neuronal apoptosis [17,84], improving hippocampal CA1 neurons [88], enhancing antioxidant capacity [17,40,85,87,89].

The aqueous extract of CSR can also improve the energy metabolism of liver mitochondria in aging model KM mice. It can also alleviate free radical damage to mitochondrial membrane structure and function and play a role in delaying aging [90].

These findings not only reveal the potential of the polysaccharide and extract in combating aging but also lay the groundwork for future clinical research. It would be beneficial to further investigate the chemical composition of CSR and the mechanism underlying anti-aging as well as their safety and effectiveness to offer novel insights and possibilities for delaying the aging process in the future (Table S3, Supplementary Materials).

### 6.4. Anti-Fatigue and Anti-Hypoxia Activities

The aqueous extract and ethanol extract of CSR are responsible for its anti-fatigue properties. By lowering the lactate index [91], inhibiting amino acid protein breakdown, and increasing glycogen reserves [92,93,94], they can improve energy metabolism. It also possesses the ability to increase the level of cyclic adenosine monophosphate (cAMP), reduce cyclic adenosine monophosphate/cyclic guanosine monophosphate (cAMP/cGMP) ratio [18], improve free radical metabolism [94,95]. In addition, CSR flavonoids (CSF) reduce MAO activity and reactive oxygen species (ROS) levels by improving free radical metabolism [96,97,98].

Oxygen deficiency can cause abnormal tissue metabolism, function, and morphology. The main cause of death is hypoxia of the brain and heart. CSR aqueous extract has positive atmospheric pressure anti-hypoxia and anti-acute cerebral ischemia and hypoxia effects [99], which increases blood hemoglobin content and enhances oxygen-carrying function [100]. As well as reducing brain edema, it increases myocardial protein content [101].

CSR exhibits remarkable anti-fatigue and anti-hypoxia properties. Research on anti-fatigue effects focuses on its active ingredients, such as water extract, ethanol extract, and flavonoids. Currently, hypoxic resistance studies are primarily focused on CSR water extract. Developing highly potent and pharmaceutically viable compounds from CSR for anti-fatigue and anti-hypoxia purposes will require further investigation (Table S4, Supplementary materials).

### 6.5. Effects on Nervous System

Ethyl acetate extract and methanol extract are both effective against A*β*_25–35_, hypoxanthine/xanthine oxidase (HPX/XO) [102], Xanthine dehydrogenase/xanthine oxidase (XDH/XO) [72] induced SK-N-SH cells have protective effects. Among them, ethyl acetate extract is more effective against Amyloid*β*-Protein 25–35 (A*β*_25–35_) and has an anti-Starosporin-induced injury effect [73]. CSP and ethyl acetate extraction sites of CSR can protect PC12 cells against damage by H_2_O_2_ [103] and A*β*_25–35_ [104]. Ethyl acetate extract has cytotoxicity to Neuro2A cells (EC_50_ = 116 mg/L) and increases the expression of synaptophysin through the mitogen-activated protein kinases (MAPK) pathway [105]. In another study, the methanol extract of CSR inhibits A*β*_25–35_ induced phosphorylation of dynamin-related protein 1 (Drp1) at Ser637 in HT22 cells and reduced the expression of Fission 1 Protein (Fis1) in H_2_O_2_ induced model for the treatment of Alzheimer’s disease (AD) [106].

Based on neuroprotective effects at the cellular level, scholars have further explored them through animal models. The ethyl acetate fraction of CSR improves the behavior of C57BL/6 male mice by reducing mitochondrial dynamics imbalance. It also downregulated the expression of the Drp1 protein and upregulated the expression of Optic Atrophy 1 (OPA1) and Mito Fusin 1 (MFN1) proteins [107]. It also improves the spatial memory and learning ability of AD model mice by regulating fecal microbiota disorder [108]. In the ovariectomized Sprague–Dawley (SD) rat model, it increased the expression of Growth-Associated Protein 43 (GAP-43) protein in the hippocampus [109], regulated the MAPK pathway, increased the expression of phosphorylation-cAMP response element-binding protein (p-CREB), and decreased the expression of p38, thereby promoting the survival and repair of hippocampal neurons.

Other studies have shown that CSR ethyl acetate extract increases the expression levels of synaptic plasticity-related proteins Syn and postsynaptic density protein-95 (PSD-95) [110] while upregulating the protein expression levels of phosphor-extracellular regulated protein kinases 1/2 (P-Erk1/2) and P-CREB in the MAPK/ERK1/2 signaling pathway [111]. It increases the effect of Long-term Potential (LTP) in Morris water maze and neuroelectrophysiology, further improving cognitive dysfunction in chronic stress Institute of Cancer Research (ICR) mice after ovariectomy [112].

The ethanol extract of CSR increased cAMP response element-binding protein /Brain-Derived Neurotrophic Factor (CREB/BDNF) expression in ovariectomized SD rats by inhibiting the p38MAPK/ERK pathway [113]. It also reduced serum corticosterone levels, increased the expression of BDNF mRNA in this region, promoted the proliferation of mouse dentate gyrus cells and differentiation of neuroblasts, enhanced the potential for hippocampal plasticity in male C57BL/6J mice [114], and thus achieved neuroprotective effects on the nerves.

The aqueous extract of CSR has a significant improvement effect on the learning and memory of scopolamine-induced KM male mice. Its mechanism may be related to reducing oxidative stress in brain tissue [115].

In Wistar male rats, through upregulating the Brain-Derived Neurotrophic Factor/Tyrosine Kinase receptor B (BDNF/TrkB) signaling pathway, enhancing cognitive function, increasing acetylcholine (ACH) content in the central cholinergic system, inhibiting cell apoptosis, and enhancing synaptic plasticity, CSF improves the AD model induced by A*β*_1–42_ [116]. In addition, CSF inhibits oxidative stress and inflammatory reactions. It can also downregulate the expression of nicotinamide adenine dinucleotide phosphate (NADPH) oxidase, ROS, and NOD-like receptor thermal protein domain associated protein 3 (NLRP3) in the hippocampus, exerting neuroprotective effects [117].

The main component of CSR, ursolic acid, at a concentration of 5–15 µM, can effectively protect SD rat hippocampal neurons from damage induced by kainic acid by regulating *α*-amino-3-hydroxy-5-methy1-4-isoxazole propionic acid (AMPA) receptors, protecting mitochondria, and reducing free radical generation [118,119].

In summary, the neuroprotective active ingredients are mainly concentrated in the methanol, ethanol, and ethyl acetate extracts of CSR. However, the main components that play a key neuroprotective role are not yet clear because of the complex components in CSR extract. Therefore, future studies should explore compounds that play a major role in protecting the nervous system (Table S5, Supplementary Materials).

### 6.6. Effects on Reproductive System

In geriatrics, benign prostatic hyperplasia (BPH) is a common genitourinary disorder characterized by prostate gland enlargement and urinary dysfunction [120]. There is an inhibitory effect of ethanol extract of CSR (2.5 mg/mL) on testosterone 5*α*-reductase [121]. Moreover, it interferes with estrogen/androgen signals to inhibit prostate hyperplasia in Wistar rats [58] and improves the disorder of prostate epithelial cells and abnormal proliferation of connective tissue in Wistar rats with BPH model, inhibiting Proliferating Cell Nuclear Antigen (PCNA), Androgen Receptor (AR), and estrogen receptor *α* (Er*α*) Protein expression while promoting estrogen receptor *β* (ER*β*) Protein expression while promoting ER*β* Protein expression [122]. Meanwhile, Wistar male rat protein expression of prostate AR, ER*α*/*β,* and 3-oxo-5-alpha-steroid 4-dehydrogenase 1/2 (SRD5A1/2) were regulated to inhibit BPH [123]. Additionally, CSR aqueous extract inhibits prostate hyperplasia, increases SOD and glutathione (GSH) activities, decreases malondialdehyde (MDA) content, and significantly reduces prostate wet weight and prostate index by improving testosterone propionate-induced oxidative stress levels [124].

In vitro experiments, Luteolin, Gallic acid, Ferulic acid, Protocatechualdehyde from CSR suppressed BPH by downregulating the expression of AR and ER*α* in BPH-1 cells and upregulation ER*β* expression [123]. CSRs containing luteolin, epicatechin, and epicatechin gallate all improve the contractility of Wistar male rats’ bladder detrusors [125].

Infertility in men is complex and multifactorial, with idiopathic infertility accounting for approximately 30% of cases [126]. It has been shown that CSR improves sexual hormone levels as a kidney tonifying traditional Chinese medicine [113]. Under the intervention of CSR aqueous extract, serum testosterone and Follicle-stimulating Hormone (FSH) levels are reduced, and interstitial cell-stimulating hormone (ICSH) levels are increased to directly affect the spermatogenic effect of immature seminiferous tubules of Wistar rats [127]. It can also promote the secretion of testosterone in SD rats and inhibit abnormal secretion of FSH and Luteinizing hormone (LH) by regulating gonadal hormone levels [128]. Glial Cell Line-derived Neurotrophic Factor (GDNF) production in testes of SD rats and undifferentiated spermatogonia proliferation stimulates, increases testosterone levels, and improves sperm motility [129]. Relieving sperm damage and serum testosterone levels are increased through the MAPK-3-mediated GDNF signaling pathway, thereby enhancing sperm motility [130].

In addition, enhancing sperm production in Wistar rats and upregulating the expression pathway of GDNF in the testes to improve male fertility [131], enhancing sperm production in golden hamsters, and blocking the impact of short photoperiod on reproductive function [132] by CSR aqueous extract.

In summary, active compounds from CSR that inhibit BPH mainly exist in its ethanol extract, while the active ingredients that promote spermatogenesis are mainly concentrated in the aqueous extract, which has been proven to have good therapeutic effects in treating male infertility (Table S6, Supplementary Materials).

### 6.7. Anti-Virus

Despite a significant increase in the number of approved antiviral drugs, these existing drugs are not always effective or well tolerated. It is becoming increasingly common for viruses to develop drug resistance. As of now, many polysaccharides have been approved as drugs as independent or major bioactive components [133]. The methyl thiazolyl tetrazolium (MTT) method was used to detect the toxicity of CSP on MT-4 cells, which showed that only sulfated polysaccharides (SCSP-M, SCSP-1, SCSP-2) are anti-HIV. Due to the interaction between sulfated polysaccharides and poly L-lysine, sulfated polysaccharides have antiviral properties [134].

In addition to the CSP, the triterpenoids contained in CSR also have antiviral activity. Ursolic acid, half ursolic malonate, malonyl oleanolic acid hemiester [10], acetyl ursolic acid, and condensed tannin extracted from CSR all have the function of inhibiting human immunodeficiency virus (HIV) protease [16]. Furthermore, triterpenoids in CSR also have inhibitory activity against hepatitis C virus (HCV) protease, with malonyl ursolic acid hemiester having the maximum inhibitory effect [47].

The main components of CSR are polysaccharides and triterpenoids, which are potentially useful for developing antiviral drugs. Additionally, it is worth noting that the antiviral efficacy of CSR has predominantly been tested in vitro with limited reports on its in vivo effects. Consequently, the precise mechanism by which CSR is antiviral remains unclear. Future studies should explore this aspect further to uncover the antiviral mechanism of CSR and establish solid foundations for its application (Table S7, Supplementary Materials).

### 6.8. Anti-Diabetic Properties

CSP as one of the pivotal active constituents in CSR, exhibits significant therapeutic effects on several diseases. Consequently, CSR is being considered a potential candidate for the development of novel anti-diabetic drugs [135]. Oral administration of CSR water-soluble polysaccharide (CSPA) significantly reduced the blood glucose level, glutamic oxaloacetic transaminase, glutamic pyruvic transaminase, blood urea nitrogen, creatinine activity in streptozotocin (STZ) induced diabetes model rats, effectively increased the serum insulin level and liver glycogen content and promoted the recovery of pancreatic islet cells in the pancreas to near normal levels [53]. CSP (300 mg/kg) can upregulate the expression of protein kinase B (AKT) and endothelial nitric oxide synthase (eNOS), and downregulate tumor necrosis factor *α* (TNF-*α*) expression [136]. It can also regulate phospholipid metabolism, including phosphatidylcholine, Lys phosphatidylcholine, phosphatidylethanolamine, and sphingomyelin to play a role in the treatment of diabetes [137].

In addition to polysaccharides, the flavonoids and their amino acid derivatives contained in CSR can also exert hypoglycemic effects. Flavan-3-ol derivatives prepared from CSR and other reagents, including 3 cysteine conjugates and 3 acetylcysteine conjugates, were found to have significant effects on α-glucosidase, sucrase, and maltase have inhibitory effects [64]. Furthermore, the flavane-3-ol oligomer and compound Pentamers (pentamer) in the stem have inhibitory effects on α-Glucosidase has inhibitory effects [138].

The investigation of CSR’s anti-diabetic activity is limited to in vitro and in vivo experiments. It plays an anti-diabetes role by regulating blood sugar levels, improving insulin sensitivity, protecting islet cells, controlling the risk of complications, etc. While these studies have demonstrated some anti-diabetic effects of CSR, further clinical trials are necessary to confirm its efficacy and safety for human use (Table S8, Supplementary Materials).

### 6.9. Anti-Osteoporosis Effect

A few studies have demonstrated the favorable anti-osteoporotic effects of CSR. After screening the methanol and water extracts of 60 natural medicinal herbs, it was found that the methanol extract of CSR has a stimulating effect on the proliferation ability of osteoblast UMR106 and an inhibitory activity on osteoclast formation [139]. CSP (100 μg/mL) induces osteogenic differentiation in MC3T3-E1 cells by activating Phosphatidylinositide 3-kinases/AKT/glycogen synthase kinase-3*β*/*β*-Catenin (PI3K/AKT/GSK3 *β*/*β*-Catenin) pathway and upregulates mRNA, PI3K, phos-pho-phosphatidylinositide 3-kinases (p⁃PI3K), AKT, phospho-protein kinase B (p⁃AKT), GSK3*β*, phosphor-glycogen synthase kinase-3*β* (p⁃GSK3*β*), *β*⁃catenin protein expression [140]. Ethanol extract of CSR can promote the differentiation of osteoblasts from MC3T3-E1 while inhibiting osteoblast apoptosis, upregulating the expression of Bax and caspase-3, and downregulating the expression of B-cell lymphoma-2 (Bcl-2) [141]. CSR aqueous extract containing serum can promote the proliferation and differentiation of MC3T3-E1 osteoblasts, increase alkaline phosphatase (ALP) activity, and increase the number of calcified nodules [142].

In in vitro experiments, CSP was administered to ovariectomized SD rats. The results express that CSP can increase the osteoclastogenesis inhibitory fac-tor/Receptor Activator for Nuclear Factor-*κ*B Ligand (OPG/RANKL) ratio, inhibit osteoclast activity by activating the OPG/Receptor Activator for Nuclear Factor-*κ*B (RANK)/RANKL signaling pathway, regulate osteocalcin levels to reduce bone turnover rate, restore the balance between bone formation and bone resorption, reduce bone loss, increase bone density, improve tibial biomechanical properties, reduce bone fragility and fracture risk, and promote osteoblast differentiation [143].

The ethanol extract of CSR can accelerate bone formation, inhibit bone resorption, and alleviate oxidative stress. It can also increase ALP levels in ovariectomized SD rats and reduce the levels of bone resorption-related biomarkers tartrate-resistant acid phosphatase (TRAP), Cathepsin K, and DPD [144]. At the same time, it can also mediate PI3K/AKT and Nuclear Factor-*κ*B (NF-*κ*B) through RANKL/RANK/ TNF receptor-associated factor 6 (TRAF6) pathway to play an anti-osteoporosis role [145].

To summarize, the anti-osteoporotic effect of CSR is primarily attributed to its extract and polysaccharide constituents. By increasing bone density, slowing down the process of osteoporosis, enhancing the resistance to fractures, reducing the risk of fractures, improving blood circulation, and increasing the nutrient supply of bones, it plays its role. However, there are currently no mechanisms of action for specific components. Further investigations are still required to clarify the underlying anti-osteoporosis mechanisms associated with the active constituents of CSR (Table S9, Supplementary Materials).

### 6.10. Liver Protection

Among its many functions, the liver plays a crucial role in immunity, metabolism, detoxification, and digestion. Fibrosis of the liver is an injury-repair response, which can be partially reversed. However, persistent damage can lead to chronic inflammation, which triggers the formation of liver fibers [146].

In order to effectively treat patients with chronic liver disease, liver fibrosis must be halted or slowed down [147]. Blood levels of glutamic oxalate transaminase (GOT) and glutamic pyruvate transaminase (GPT) increase when the liver is damaged. In liver injury induced by Streptozocin (STZ) in Wistar rats, CSPA (200 mg/kg, 150 mg/kg) reduces levels of GOT and GPT [53]. By increasing white blood cell (WBC) levels, hematocrit (HCT) levels, red blood cells (RBCs), mean corpuscular volumes (MCVs), and red blood cell distribution width (RDW) levels in the blood cells of SD male rats induced by carbon tetrachloride. CSR extract regulates the transforming growth factor *β*1 (TGF-*β*1) expression [148] and increases levels of WBC, HCT, RBC, MCV, and RDW in the blood cells of SD male rats induced by carbon tetrachloride, to impact blood cell typing and alleviate symptoms of liver fibrosis [149]. Furthermore, it can also reduce the liver’s exposure to the inflammatory factors TGF-*β*1, TNF-*α,* and interleukin 1 (IL-1) stimulation, thereby reducing liver fibrosis [150]. CSR aqueous extract (3.5 g/kg) alleviates the lipid peroxidation damage caused by free radicals attacking the liver cell membrane of male Wister rats and protects the liver tissue from normal physiological operation [92].

The use of 60% ethanol extract from CSR has been found to reduce serum levels of aspartate aminotransferase (AST), alanine aminotransferase (ALT), lactate dehydro-genase (LDH), and laminin (LN) in KM male mice induced by carbon tetrachloride. Additionally, the extract of CSR reduced the content of Hyp and MDA in liver tissue, while increasing SOD and GSH. By increasing the body’s antioxidant level and scavenging free radicals, reducing collagen fiber production, and reducing extracellular matrix deposition, the extract of CSR protects the liver [151].

HCY2 and ursolic acid isolated from the ethanol extract of CSR can enhance mitochondrial function and glutathione antioxidant status in liver tissue, inhibit plasma aspartate aminotransferase (AST) and alanine aminotransferase (ALT) activities, and protect SD female rats from carbon tetrachloride damage [152]. CSF enhances the activity of SOD and Glutathione peroxidase (GSH-Px) in formaldehyde-induced T6 cells [153], reduces the protein concentration and MDA content of H_2_O_2_-induced damage to T6 cells, increases the expression level of nitric oxide synthase (NOS) protein [154], and has a protective effect on oxidative damage to T6 cells.

Along with the current increasing demand for hepatoprotective drugs, CSR has demonstrated promising potential in the field of drug development for liver protection. However, due to the intricate physiological functions of the liver, further investigations are required to elucidate more specific mechanisms underlying hepatoprotection. Additionally, it is also imperative to conduct comparative analyses of CSR constituents to assess their respective hepatoprotective abilities (Table S10, Supplementary Materials).

### 6.11. Other Pharmacological Effects

#### 6.11.1. Intestinal Effects

A specific effect of CSR is to promote intestinal peristalsis, facilitate bowel movement, and maintain intestinal moisture. CSP (14.28 mg/kg, 28.57 mg/kg, 57.14 mg/kg) counteracted atropine’s inhibitory effect on intestinal peristalsis in KM mice by modulating parasympathetic nervous system function, reducing phenol red residue, and increasing intestinal propulsion rates [155]. Comparing the effects of aqueous extract with ethyl acetate, methanol, and the aqueous extraction site of CSR on intestinal defecation in KM mice, it was observed that the aqueous extract (3.9 g/kg) showed significant activity [43]. As demonstrated by the aqueous extracts of CSR (0.01 g/mL, 0.015 g/mL, 0.02 g/mL), the CSR augments smooth muscle contraction frequency while attenuating smooth muscle contraction amplitude in New Zealand white rabbits, resulting in mild “intestinal moistening and purging” effects [156].

#### 6.11.2. Mitigate Obesity

Ursolic acid (UA), an active component of CSR, HCY2 significantly reduced both body weight gain and fat pad weight in ICR mice [157]. Furthermore, the expression of mitochondrial uncoupling protein 3 in skeletal muscle can be increased by ursolic acid through the regulation of the Adenosine phosphate-activated protein kinase/peroxisome proliferator-activated receptor *γ* coactivator-1 (AMPK/PGC1) pathway, thereby potentially contributing to the treatment of obesity [158].

#### 6.11.3. Renal Protective Effects

With the aggravation of diabetes and the side effects of hypoglycemic drugs, kidney damage is gradually caused. Serum levels of blood urea nitrogen (BUN) and creatinine (Cr) are significantly increased, which is considered to be an important indicator of renal dysfunction. HCY2 (0.5 mg/kg, 1.0 mg/kg) and ursolic acid (0.35 mg/kg, 0.70 mg/kg), derived from the CSR, resulted in a reduction in BUN and Cr levels and provided protection against gentamicin-induced nephrotoxicity in female SD rats [152]. CSPA (200 mg/kg, 150 mg/kg) in vivo significantly reduces the levels of BUN and Cr, thereby ameliorating renal dysfunction in streptozotocin-induced Wistar rats [53]. The CSP concentrations (0.25 mg/mL, 0.5 mg/mL, 1.0 mg/mL) indirectly attenuated H_2_O_2_-induced apoptosis of VERO cells by suppressing caspase-3 activity in vitro, indicating the potential of CSR for the prevention and treatment of kidney-related diseases [159].

In summary, CSR protects kidney function from further damage by improving renal blood circulation, anti-fibrotic, antioxidant, and anti-inflammatory effects. For acute kidney injury, renal protection can promote the repair and regeneration of kidney tissue, reduce oxidative damage and inflammatory reactions, and help alleviate the degree of kidney injury and restore kidney function. For chronic renal failure, renal protection can delay the progression of the disease and reduce the loss of renal function.

#### 6.11.4. Immune System Modulation

Studies have demonstrated that CSR exine levels effectively inhibit the autoimmune antibodies and enhance humoral immune function, thereby improving overall immune competence in the body.

The 75% alcohol extract (0.1 g/kg, 0.2 g/kg, 0.4 g/kg) and aqueous extract (0.18 g/kg, 0.36 g/kg, 0.72 g/kg) of CSR significantly augmented the thymus index and spleen index in immunosuppressed KM mice while also enhancing phagocytic function within the immune system. They promoted hemolysin antibody production and increased serum levels, interferon-*γ* (IFN-*γ*), and TNF-*α* secretion, thereby bolstering both humoral and cellular immunity responses; notably, aqueous extract to the ethanol extract [14]. The aqueous extract of CSR part Ⅲ (300 mg/kg) demonstrated a protective effect on BALB/C mice immunosuppressed by cyclophosphamide (CTX). It enhanced the phagocytic capacity of macrophages towards foreign bodies and resulted in an elevation in serum, effectively improving the humoral immune function of mice [160]. In addition to the immunomodulatory effects observed with CSR aqueous extract and ethanol extract, CSP exhibits significant immunomodulatory effects in vitro experiments. Specifically, CSP polysaccharide demonstrates remarkable potential as it promotes the proliferation and enhances the phagocytic activity of RAW264.7 macrophages at concentrations ranging from 25 to 400 μg/mL. Moreover, CSP also induces an increase in the secretion levels of IL-6, TNF-α, and NO [161].

#### 6.11.5. Anti-Ulcer Effect

In recent years, despite the efficacy of antiplatelet drugs such as aspirin and clopidogrel in managing arterial circulation disorders caused by excessive platelet aggregation, it is crucial to consider potential gastrointestinal complications like gastric bleeding and ulceration when administering these medications.

CSR has also demonstrated positive outcomes in the restoration and optimization of digestive functionality. The following examples are provided. The administration of CSP (100 mg/kg, 200 mg/kg, 400 mg/kg) effectively inhibits the development of water immersion restraint stress-induced gastric ulcers and pyloric ligation-induced gastric ulcer index in Wister rats. It also enhances the microcirculation of the gastric mucosa and improves its defensive capabilities, thereby exerting an anti-ulcer effect [162]. Additionally, CSR can stimulate the synthesis and release of endogenous prostaglandin E2 (PGE2) and epidermal growth factor (EGF), enhance mucosal blood defense and repair functions of gastric mucosa, suppress the inflammatory mediator platelet-activating factor (PAF), mitigate its damage to mucosa, and restore the balance and defense factors for achieving an anti-gastric ulcer effect [163].

#### 6.11.6. Anti-Depressant Effect

The therapeutic potential of CSF has garnered significant attention in research studies. The administration of CSF at doses of 0.2 g/kg, 0.1 g/kg, and 0.05 g/kg has mitigated perimenopausal depression in female SD rats by modulating the hypothalamic-pituitary-gonadal axis through an increase in E2 levels [164]. Not singly but in pairs, CSF (400 mg/kg, 200 mg/kg, 100 mg/kg) also effectively demonstrates significant therapeutic efficacy in perimenopausal depression KM female mice, ameliorating the pathological alterations in the uterus, thymus, spleen, and hypothalamus [165].

#### 6.11.7. Anti-Epileptic

The maximum electroconvulsive seizure (MES) model is widely regarded as a robust experimental model for grand mal epilepsy. The clinical efficacy of drugs with potent anti-MES effects extends to grand mal seizures. Based on this, CSR aqueous extract (1 g/mL), which exhibits a potent anti-MES effect in KM mice, holds promising potential for the treatment of grand mal epilepsy [99].

#### 6.11.8. Anti-Bacterial

For good measure, the polyphenolic compounds and polymeric procyanidins present in CSR exhibit antibacterial properties. Cynomoriitannin (MIC = 64 μg/mL) demonstrates higher efficacy against methicillin-resistant staphylococcus aureus (MRSA) than other compounds separated from CSR [41] (Table S11, Supplementary Materials).

## 7. Toxicity

A growing awareness of food safety has led to a growing focus on CSR, which is a homology between medicine and food. The evaluation of a new potential drug’s safety is crucial not only in the concept of fitness and healthcare but also in its research and development. Several studies were conducted to evaluate the safety of CSR, including the contents of heavy metal ions detection and toxicity studies in vivo.

### 7.1. Heavy Metal Ions Detection

The levels of Cu, Pb, Cd, Cr, As, and Hg in CSR have been determined by microwave digestion and high-resolution continuous light source atomic absorption spectrometry [166]. The results indicated that the concentrations of these metals in CSR were far below the limitations of both the Green Industry Standard for Importing Medicinal Plants and Preparations as well as the national food safety standard named Maximum Levels of Contaminants in Foods (GB2762–2012).

### 7.2. Toxicity Studies In Vivo

Currently, CSR aqueous extract has been confirmed to have no obvious toxicity by experiments on acute toxicity tests, teratogenicity tests, and subchronic toxicity tests.

In acute toxicity experiments, the oral LD_50_ of CSR aqueous extract exceeded 21.5 g/kg in all cases [167]. The results of another study indicated that the maximum tolerable dose of CSR in KM mice is greater than 15 g/kg [168], also providing evidence of CSR aqueous extract nonobvious toxicity.

*Salmonella typhimurium* reverse mutation test, bone marrow polychromatic red blood cell micronucleus test, and sperm aberration test in KM mice were adopted to further investigate the genetic toxicity of CSR aqueous extract. One of the studies indicated that CSR aqueous extract (7.5 g/kg, 3.75 g/kg, and 1.875 g/kg) could not induce tested strains (TA97, TA98, TA100, TA102) to form colonies, as well as did not cause any mutagenic effects against the somatic cells and germ cells [169]. The results of the experiment are proved by another study conducted with CSR aqueous extract (2.25, 4.50, 9.00 g/kg), with the difference being the type of experimental strains (TA97, TA98, TA100, TA102, and TA1535) [167].

As the third stage of food safety toxicological evaluation, a subchronic toxicity test was conducted for a ninety-day feeding trial. No significant toxicological findings were detected in hematological parameters or clinical and pathological examinations when feeding various concentrations of CSR aqueous extract (1.04 g/kg, 2.08 g/kg, 4.16 g/kg) to SD rats [168]. Three Doses of CSR aqueous extract (2.83 g/kg, 5.66 g/kg, 8.49 g/kg) were fed to Wistar rats in the same year’s research. Fu. Et [170] found that the medium (5.66 g/kg) and high (8.49 g/kg) dose groups exhibited significantly increased plasma prothrombin time (PT), as well as testicular organ coefficient and epididymal organ coefficient. The maximum no-observed-adverse-effect level (NOAEL) was determined to be 2.83 g/kg, while the minimum lowest-observed-adverse-effect level (LOAEL) was identified at 5.66 g/kg in this subchronic transoral toxicity study.

However, according to clinical reports, a patient was diagnosed with acute renal function injury after taking a single Chinese medicine CSR 100–150 g aqueous extract for about 0.5 h, experiencing nausea and vomiting 4–6 times, non-jet like, with all vomit being gastric contents, without abdominal pain or diarrhea, headache, or fever. Therefore, it is still essential to exercise caution and avoid an overdose of CSR [167].

Based on the current situation, most studies about the toxicity of CSR focus on its aqueous extract, and few on other extracts and extract components. In order to build a more comprehensive toxicity evaluation, subsequent studies are required to focus on CSR extracts from other solvents. Furthermore, further exploration of toxic compounds in CSR is also necessary. These will provide a more reliable scientific basis for the safe use of CSR.

## 8. Conclusions

In recent years, due to China’s aging population and growing demand for healthcare, CSR has emerged as a highly valuable Chinese herbal medicine. It is currently being investigated for its medicinal properties. A few studies have highlighted the beneficial effects of CSR on overall health. This has led to its incorporation into various compound preparations and health supplements, expanding its potential applications [171].

This study summarizes various studies on *Cynomorium songaricum* Rupr. (CSR) from various aspects such as botany, ethnic pharmacology, phytochemistry, modern pharmacology, and toxicology. Compared with previous reviews describing the effects of CSR, the data on phytochemistry and pharmacology in this study are complete and more comprehensive, which helps to provide a data reference for professionals studying CSR.

Traditional Chinese medicine CSR remains an outstanding kidney yang and tonifying remedy. Additionally, it nourishes the essence and blood, as well as moistening the intestines and bowel movements. Consequently, several traditional CSR prescriptions have been included in the Chinese Pharmacopoeia as modern clinical medications. Various active compounds such as flavonoids, terpenoids, and polysaccharides may be the molecular basis for CSR pharmacological activity. A portion of the 98 compounds isolated from CSR have pharmacological properties, including anti-tumor, antioxidant, neuroprotective, antiviral, and anti-diabetic properties, etc. However, most studies involving CSR mainly focus on simple validation of its effectiveness in extract administration. In vitro studies rarely involve in vivo mechanisms and there is a lack of modern pharmacological mechanism research on traditional Chinese medicine compound formulations. 

Looking back at the entire article, there are also some shortcomings in this study. The literature collection work is up to June 2023. Afterwards, more recently published CSR-related studies may not be included. Although CSR has become a well-known health medication in China, its popularity internationally is still insufficient. This may be related to its limited distribution in other countries. Therefore, it is inevitable that foreign research cited is relatively scarce. We hope that this study can attract more people to be interested in and involved in the study of CSR. Furthermore, we look forward to the future where traditional Chinese medicine of this kind will be included in foreign pharmacopoeias. Furthermore, in phytochemistry, there is a lack of research on the pharmacological activities of some compounds isolated from CSR, so their IC50 values were not included in this study.

In a nutshell, the future direction of CSR should focus on further research into the pharmacology and toxicity of compounds. Further exploration of the pharmacological mechanisms underlying CSR needs to be conducted to address the current lack of research data, as well as the development of functional health products.

## Figures and Tables

**Table 2:** Flavonoids isolated from *Cynomorium songaricum* Rupr.

No.	Compound	Parts of Plant	Extract Solvent	Identification	References
**1**	Phloridzin	Stems	70% acetone	HPLC, ^1^H NMR, ^13^C NMR	[42]
**2**	(−)-Epicatechin	Stems	chloroform	UV, MS, ^1^H NMR, ^13^C NMR	[9]
**3**	Naringenin	Stems	chloroform	TLC, UV, IR, ^1^H NMR, ^13^C NMR	[9]
**4**	(−)-Catechin	Stems	ethyl acetate	IR, ESI-MS, ^1^H NMR, ^13^C NMR	[43]
**5**	Luteolin-7-*O*-glucoside	Stems	ethyl acetate part	^1^H NMR, ^13^C NMR	[16]
**6**	Procyanidin B1	Stems	aqueous	^1^H NMR, ^13^C NMR	[16]
**7**	Procyanidin B6	Stems	aqueous	^1^H NMR, ^13^C NMR	[16]
**8**	Procyanidin B3	Fresh stems	70% acetone	HPLC, ^1^H NMR, ^13^C NMR	[41]
**9**	Catechin-(6′-8)-catechin	Fresh stems	70% acetone	HPLC, ^1^H NMR, ^13^C NMR	[41]
**10**	Catechin-(6′-6)-catechin	Fresh stems	70% acetone	HPLC, ^1^H NMR, ^13^C NMR	[41]
**11**	Epicatechin-(4*β*-8)-epicatechin-(4*β*-8)-catechin	Fresh stems	70% acetone	HPLC, ^1^H NMR, ^13^C NMR	[41]
**12**	Epicatechin-(4*β*-6)-epicatechin-(4*β*-8)-catechin	Fresh stems	70% acetone	HPLC, ^1^H NMR, ^13^C NMR	[41]
**13**	Arecatannin A1	Fresh stems	70% acetone	HPLC, ^1^H NMR, ^13^C NMR	[41]
**14**	Dehydrodiconiferyl alcohol-9-*O*-*β*-D-glu-copyranoside	Fresh stems	ethyl acetate part	^1^H NMR, ^13^C NMR	[4]
**15**	3′,4′,5,7-tetrahydroxy-flavanone-2(S)-3′-*O*-*β*-D-glucopyranoside	Fresh stems	ethyl acetate part	^1^H NMR, ^13^C NMR	[4]
**16**	Luteolin-4′-*O*-*β*-glucopyranoside	Fresh stems	ethyl acetate part	^1^H NMR, ^13^C NMR	[4]
**17**	Astragalin	Fresh stems	ethyl acetate part	^1^H NMR, ^13^C NMR	[4]
**18**	Quercetin-3-*O*-rutinoside	Fresh stems	ethyl acetate part	^1^H NMR, ^13^C NMR	[4]
**19**	Naringenin-7-*O*-*β*-D-glucopyranoside	Fresh stems	ethyl acetate part	^1^H NMR, ^13^C NMR	[4]
**20**	Naringenin-5-*O*-*β*-D-glucopyranoside	Fresh stems	ethyl acetate part	^1^H NMR, ^13^C NMR	[4]
**21**	Naringenin-4′-*O*-*β*-pyranoglucose	Whole grass	*N*-butanol part	^1^H NMR, ^13^C NMR	[44]
**22**	Cyanidin 3-*O*-glucoside	Inflorescences	95% alcohol	^1^H NMR, ^13^C NMR	[45]
**23**	Cyanidin 3-*O*-rhamnosylglucoside	Inflorescences	95% alcohol	^1^H NMR, ^13^C NMR	[45]
**24**	(+)-Catechin	Inflorescences	95% alcohol	TLC, ^1^H NMR, ^13^C NMR	[46]
**25**	Isoquercetin	Inflorescences	95% alcohol	^1^H NMR, ^13^C NMR	[46]
**26**	Rutin	Inflorescences	95% alcohol	^1^H NMR, ^13^C NMR	[46]
**27**	(−)-Epicatechin-3-*O*-gallate	Inflorescences	95% alcohol	TLC, ^1^H NMR, ^13^C NMR	[46]

UV: Ultraviolet spectrophotometry; IR: Infrared spectroscopy; ESI-MS: Electrospray ionization mass spectrometry; ^13^C NMR: Carbon-13 nuclear magnetic resonance spectrometry; ^1^H NMR: Hydrogen-1 nuclear magnetic resonance spectrometry; HPLC: High-pressure liquid chromatography; TLC: Thin layer chromatography.

**Table 3:** Terpenoids isolated from *Cynomorium songaricum* Rupr.

No.	Compound	Parts of Plant	Extract Solvent	Identification	References
**28**	Malonyl ursolic acid hemiester	Stems	dichloromethane	^1^H NMR, ^13^C NMR	[16]
**29**	Ursolic acid	Stems	dichloromethane	^1^H NMR, ^13^C NMR	[16]
**30**	Acetyl ursolic acid	Stems	dichloromethane	^1^H NMR, ^13^C NMR	[16]
**31**	Oleanolic acid	Stems	dichloromethane	IR, ^1^H NMR, ^13^C NMR, HR-MS	[16]
**32**	Betulinic acid	Stems	dichloromethane	^1^H NMR, ^13^C NMR	[16]
**33**	Malonyl oleanolic acid hemiester	Stems	dichloromethane	HPLC, ^1^H NMR, ^13^C NMR	[10]
**34**	Glutaryl ursolic acid hemiester	Stems	ethyl acetate	HPLC-MS	[47]
**35**	Oxalyl ursolic acid hemiester	Stems	ethyl acetate	HPLC-MS	[47]
**36**	Succinyl ursolic acid hemiester	Stems	ethyl acetate	HPLC-MS	[47]
**37**	Ursolic acid methyl ester	Stems	ethyl acetate	HPLC-MS	[47]
**38**	3*β*,28-Dihydroxyoleana-11,13(18)-diene	Stems	ethyl acetate part	^1^H NMR, ^13^C NMR	[48]
**39**	Maslinic acid	Stems	aqueous	ESI-MS, ^1^H NMR, ^13^C NMR	[49]

IR: Infrared spectroscopy; ^13^C NMR: Carbon-13 nuclear magnetic resonance spectrometry; ^1^H NMR: Hydrogen-1 nuclear magnetic resonance spectrometry; HR-MS: High-resolution mass spectrometry; ESI-MS: Electrospray ionization mass spectrometry; HPLC: High-pressure liquid chromatography; HPLC-MS: High-performance liquid chromatography-mass spectrometry.

**Table 4:** Steroids isolated from *Cynomorium songaricum* Rupr.

No.	Compound	Parts of Plant	Extract Solvent	Identification	References
**40**	5*α*-Stigmast-9(11)-en-3*β*-ol	Stems	ethyl acetate	HR-MS, ^1^H NMR, ^13^C NMR	[12]
**41**	5*α*-Stigmast-9(11)-en-3*β*-ol tetracosatrienoic acid ester	Stems	ethyl acetate	HR-MS, ^1^H NMR, ^13^C NMR	[12]
**42**	Daucosterol	Stems	ethyl acetate part	TLC	[13]
**43**	*β*-Sitosterol	Stems	ethyl acetate part	TLC	[13]
**44**	*β*-Sitosteryl oleate	Stems	dichloromethane	HPLC, ^1^H NMR, ^13^C NMR	[16]
**45**	*β*-Sitosteryl glucoside	Stems	dichloromethane	HPLC, ^1^H NMR, ^13^C NMR	[16]
**46**	*β*-Sitosteryl glucoside 6′-*O*-aliphatates	Stems	dichloromethane	HPLC, ^1^H NMR, ^13^C NMR	[16]
**47**	*β*-Sitosterol palmaitate	Stems	chloroform	HPLC, ^1^H NMR, ^13^C NMR	[50]
**48**	Campesterol	Stems	petroleum ether	GC-MS	[51]
**49**	*γ*-Sitosterol	Stems	petroleum ether	GC-MS	[51]

HR-MS: High-resolution mass spectrometry; HPLC: High-pressure liquid chromatography; TLC: Thin layer chromatography; ^13^C NMR: Carbon-13 nuclear magnetic resonance spectrometry; ^1^H NMR: Hydrogen-1 nuclear magnetic resonance spectrometry; GC-MS: Gas chromatography–mass spectrometry.

**Table 5:** Saccharides and glycosides isolated from *Cynomorium songaricum* Rupr.

No.	Compound	Parts of Plant	Extract Solvent	Identification	References
**50**	Glucose	Stems	chloroform	TLC, GC-MS	[9]
**51**	Zingerone 4-*O*-*β*-D-glucopyranoside	Stems	dichloromethane	FAB-MS, ^1^H NMR, ^13^C NMR, HMQC, HMBC	[10]
**52**	*n*-Butyl-*β*-D-fructofuranoside	Stems	ethyl acetate	^1^H NMR, ^13^C NMR	[55]
**53**	*n*-Butyl-*α*-D-fructofuranoside	Stems	ethyl acetate	^1^H NMR, ^13^C NMR	[11]
**54**	*n-*Butyl-*β-*D-fructopyranoside	Stems	ethyl acetate	^1^H NMR, ^13^C NMR	[56]
**55**	Piceid	Stems	ethyl acetate part	^1^H NMR, ^13^C NMR	[16]
**56**	Coniferin	Stems	N-butanol part	^1^H NMR, ^13^C NMR	[16]
**57**	Isoconiferin	Stems	N-butanol part	^1^H NMR, ^13^C NMR	[16]
**58**	Adenosine	Stems	N-butanol part	HPLC, ^1^H NMR, ^13^C NMR	[16]
**59**	(−)-Isolariciresinol 4-*O*-*β*-D-glucopyranoside	Stems	aqueous	FAB-MS, CD, ^1^H NMR, ^13^C NMR	[42]
**60**	(7S,8R)-Dehydrodiconiferyl alcohol 9′-*β*-glucopyranoside	Stems	aqueous	FAB-MS, HPLC, ^1^H NMR, ^13^C NMR, CD, ^1^H–^1^HCOSY	[42]
**61**	Nicoloside	Stems	aqueous	^1^H NMR, ^13^C NMR	[42]
**62**	Songaricumone A	Fresh stems	ethyl acetate part	HR-MS, ^1^H-NMR, ^1^H–^1^HCOSY, HMBC, UV, TLC, CD	[4]

UV: Ultraviolet spectrophotometry; TLC: Thin layer chromatography; GC-MS: Gas chromatography–mass spectrometry; FAB-MS: Fast atom bombardment mass spectrometry; HMQC: Heteronuclear multiple quantum coherence; HMBC: Heteronuclear multiple bond connectivity; HPLC: High-pressure liquid chromatography; CD: Circular dichroism; ^13^C NMR: Carbon-13 nuclear magnetic resonance spectrometry; ^1^H NMR: Hydrogen-1 nuclear magnetic resonance spectrometry; ^1^H–^1^HCOSY: Homonuclear Correlation Spectroscopy.

**Table 6:** Organic acids and organic acid ester isolated from *Cynomorium songaricum* Rupr.

No.	Compound	Parts of Plant	Extract Solvent	Identification	References
**63**	Protocatechuic acid	Stems	ethyl acetate part	^1^H NMR, ^13^C NMR	[13]
**64**	Gallic acid	Stems	ethyl acetate part	^1^H NMR, ^13^C NMR	[13]
**65**	*n*-Butyric acid	Stems	ethyl acetate part	^1^H NMR, ^13^C NMR	[13]
**66**	4-Methoxycinnamic acid	Stems	ethyl acetate part	^1^H NMR, ^13^C NMR	[48]
**67**	*p*-Hydroxybenzoic acid	Stems	ethyl acetate part	^1^H NMR, ^13^C NMR	[42]
**68**	Methyl protocatechuicate	Stems	ethyl acetate part	^1^H NMR, ^13^C NMR	[42]
**69**	*p*-Hydroxycinnamic acid	Stems	ethyl acetate part	^1^H NMR, ^13^C NMR	[16]
**70**	3,4-Dihydroxy-benzoic acid ethyl ester	Stems	ethyl acetate part	^1^H NMR, ^13^C NMR	[57]
**71**	4-Hydroxyphenethyl 2-(4-hydroxyphenyl) acetate	Stems	ethyl acetate part	^1^H NMR, ^13^C NMR, HMBC, HMQC	[48]
**72**	Stearic acid *α-*monoglyceride	Stems	ethyl acetate part	ESI-MS, ^1^H NMR, ^13^C NMR	[13]
**73**	Succinic acid	Stems	aqueous part	IR, ^1^H-NMR	[43]
**74**	Ferulic acid	Stems	70% alcohol	^1^H NMR, ^13^C NMR	[58]
**75**	Gentisic acid	Stems	aqueous	^1^H NMR, ^13^C NMR	[49]
**76**	Palmitic acid	Stems	aqueous	EI-MS, ^1^H NMR, ^13^C NMR	[49]
**77**	3,4-Dihydroxyphenethyl acetate	Stems	aqueous	EI-MS, ^1^H NMR, ^13^C NMR	[49]
**78**	Vanillic acid	Whole grass	aqueous part	^1^H NMR, ^13^C NMR	[44]
**79**	Capilliplactone	Whole grass	ethyl acetate part	IR, ^1^H NMR, ^13^C NMR, ^1^H–^1^HCOSY, HMQC	[59]

HMQC: Heteronuclear multiple quantum coherence; HMBC: Heteronuclear multiple bond connectivity; ESI-MS: Electrospray ionization mass spectrometry; IR: Infrared spectroscopy; EI-MS: Electron impact mass spectrometry; ^13^C NMR: Carbon-13 nuclear magnetic resonance spectrometry; ^1^H NMR: Hydrogen-1 nuclear magnetic resonance spectrometry; ^1^H–^1^HCOSY: Homonuclear Correlation Spectroscopy.

**Table 7:** Phloroglucinol adducts isolated from *Cynomorium songaricum* Rupr.

No.	Compound	Parts of Plant	Extract Solvent	Identification	References
**80**	Epicatechin-(4*β*-2)-phloroglucinol	Fresh stems	70% acetone	HPLC-MS, HPLC	[41]
**81**	Epicatechin-3-O-gallate-(4*β*-2)-phloroglucinol	Fresh stems	70% acetone	HPLC-MS, HPLC	[41]
**82**	Catechin-(4*α*-2)-phloroglucinol	Fresh stems	70% acetone	HPLC-MS, HPLC	[41]
**83**	Cynomoriitannin-phloroglucinol A	Fresh stems	70% acetone	CD, ^1^H NMR, ^13^C NMR	[41]
**84**	Cynomoriitannin-phloroglucinol B	Fresh stems	70% acetone	CD, ^1^H NMR, ^13^C NMR	[41]
**85**	Phloroglucinol	Stems	aqueous	^1^H NMR, ^13^C NMR	[49]

HPLC-MS: High-performance liquid chromatography–mass spectrometry; HPLC: High-pressure liquid chromatography; CD: Circular dichroism; ^13^C NMR: Carbon-13 nuclear magnetic resonance spectrometry; ^1^H NMR: Hydrogen-1 nuclear magnetic resonance spectrometry.

**Table 8:** Other compounds isolated from *Cynomorium songaricum* Rupr.

No.	Compound	Parts of Plant	Extract Solvent	Identification	References
**86**	Mannitol	Stems	aqueous	^1^H NMR, ^13^C NMR	[49]
**87**	Protocatechualdehyde	Stems	70% alcohol	^1^H NMR, ^13^C NMR	[58]
**88**	Chrysophanol	Stems	70% alcohol	^1^H NMR, ^13^C NMR	[58]
**89**	Emodin	Stems	70% alcohol	^1^H NMR, ^13^C NMR	[58]
**90**	Physcion	Stems	70% alcohol	^1^H NMR, ^13^C NMR	[58]
**91**	(−)-Lariciresinol	Stems	ethyl acetate part	^1^H NMR, ^13^C NMR	[57]
**92**	4-Methylcatechol	Stems	ethyl acetate part	^1^H NMR, ^13^C NMR	[57]
**93**	4*β*-(L-cysteinyl)-catechin	Stems	70% acetone	ESI-MS, ^1^H NMR, ^13^C NMR	[64]
**94**	4*β*-(L-cysteinyl)-epicatechin	Stems	70% acetone	ESI-MS, ^1^H NMR, ^13^C NMR	[64]
**95**	4*β*-(L-cysteinyl)-epicatechin 3-*O*-gallate	Stems	70% acetone	ESI-MS, ^1^H NMR, ^13^C NMR	[64]
**96**	4*β*-(L-acetylcysteinyl)-epicatechin	Stems	95% alcohol	ESI-MS, ^1^H NMR, ^13^C NMR	[64]
**97**	4*β*-(L-acetylcysteinyl)-epicatechin 3-*O*-gallate	Stems	95% alcohol	ESI-MS, ^1^H NMR, ^13^C NMR	[64]
**98**	4*β*-(L-acetylcysteinyl)-epiafzelechin	Stems	95% alcohol	ESI-MS, ^1^H NMR, ^13^C NMR	[64]

ESI-MS: Electrospray ionization mass spectrometry; ^13^C NMR: Carbon-13 nuclear magnetic resonance spectrometry; ^1^H NMR: Hydrogen-1 nuclear magnetic resonance spectrometry.

## Data Availability

Data are contained within the article and Supplementary Materials.

## Supplementary Materials

The following supporting information can be downloaded at: https://www.mdpi.com/article/10.3390/molecules29050941/s1. Table S1. *Cynomorium songaricum* anti-tumor effects. Table S2. *Cynomorium songaricum* anti-oxidation function. Table S3. *Cynomorium songaricum* anti-aging effects. Table S4. *Cynomorium songaricum* anti-fatigue and anti-hypoxia activities. Table S5. *Cynomorium songaricum* effects on nervous system. Table S6. *Cynomorium songaricum* effects on reproductive system. Table S7. *Cynomorium songaricum* anti-virus. Table S8. *Cynomorium songaricum* anti-diabetic properties. Table S9. *Cynomorium songaricum* anti-osteoporosis effect. Table S10. *Cynomorium songaricum* liver protection. Table S11. *Cynomorium songaricum* other pharmacological effects.

## Abbreviations

CSR	*Cynomorium songaricum* Rupr.	HPLC-MS	High-performance liquid chromatography–mass spectrometry.
VU	Vulnerable	TLC	Thin layer chromatography
IUCN	International Union for Conservation of Nature	HR-MS	High-resolution mass spectrometry
CITES	Convention on International Trade in Endangered Species of Wild Fauna and Flora	GC-MS	Gas chromatography–mass spectrometry
UV	Ultraviolet spectrophotometry	FAB-MS	Fast atom bombardment mass spectrometry
IR	Infrared spectroscopy	HMQC	Heteronuclear multiple quantum coherence
ESI-MS	Electrospray ionization mass spectrometry	HMBC	Heteronuclear multiple bond connectivity
^13^C NMR	Carbon-13 nuclear magnetic resonance spectrometry	CD	Circular dichroism
^1^H NMR	Hydrogen-1 nuclear magnetic resonance spectrometry	^1^H–^1^HCOSY	Homonuclear Correlation Spectroscopy
HPLC	High-pressure liquid chromatography	CSP	CSR polysaccharides
CSF	CSR flavonoids	ABTS	2,2′-Azino-bis(3-ethylbenzothiazoline-6-sulfonic acid) diammonium salt
TERT	Telomerase reverse transcriptase	XO	Xanthine oxidase
BNIP3	Bcl-2/adenovirus E1B 19 kDa-interacting protein 3	SOD	Superoxide dismutase
BNIP3L	Bcl-2/adenovirus E1B 19 kDa protein-interacting protein 3-like	KM mice	Kunming mice
DPPH	2,2-Diphenyl-1-picrylhydrazyl	cAMP	Cyclic adenosine monophosphate
cGMP	Cyclic guanosine monophosphate	BDNF	Brain-Derived Neurotrophic Factor
MAO	monoamine oxidase	TrkB	Tyrosine Kinase receptor B
ROS	Reactive oxygen species	ACH	Acetylcholine
HPX/XO	Hypoxanthine/Xanthine oxidase	NADPH	Nicotinamide adenine dinucleotide phosphate
XDH/XO	Xanthine dehydrogenase	NLRP3	NOD-like receptor thermal protein domain associated protein 3
A*β*_25–35_	Amyloid*β*-Protein 25–35	AMPA	Amino-3-hydroxy-5-methy1-4-isoxazole propionic acid
MAPK	Mitogen-activated protein kinases	BPH	Benign prostatic hyperplasia
Drp1	Dynamin-related protein 1	PCNA	Proliferating Cell Nuclear Antigen
Fis1	Mitochondrial Fission 1 Protein	AR	Androgen Receptor
AD	Alzheimer’s disease	Er*α*	Estrogen receptor *α*
OPA1	Optic Atrophy 1	Er*β*	Estrogen receptor *β*
MFN1	Mitofusin 1	SRD5A 1/2	3-oxo-5-alpha-steroid 4-dehydrogenase 1/2
SD rat	Sprague-Dawley rat	GSH	Glutathione
GAP-43	Growth-Associated Protein 43	MDA	Malondialdehyde
p-CREB	Phosphorylation-cAMP response element-binding protein	FSH	Follicle-stimulating Hormone
PSD-95	Postsynaptic density protein-95	ICSH	Interstitial cell-stimulating hormone
p-Erk	Phosphor-extracellular regulated protein kinases	LH	Luteinizing hormone
LTP	Long-term Potential	GDNF	Glial Cell Line-derived Neurotrophic Factor
ICR	Institute of Cancer Research	MTT	Methyl thiazolyl tetrazolium
CREB	cAMP response element-binding protein	HIV	Human immunodeficiency virus
HCV	Hepatitis C virus	p-GSK3*β*	phosphor-glycogen synthase kinase-3*β*
CSPA	CSR water-soluble polysaccharide	Bcl-2	B-cell lymphoma-2
STZ	Streptozotocin	ALP	Alkaline phosphatase
AKT	American karate tae	OPG	Osteoclastogenesis inhibitory factor
eNOS	endothelial nitric oxide synthase	RANKL	Receptor Activator for Nuclear Factor-κ B Ligand
TNF-*α*	Tumor necrosis factor *α*	RANK	Receptor Activator for Nuclear Factor-κ B
PI3K	Phosphatidylinositide 3-kinases	TRAP	Tartrate-resistant acid phosphatase
p-PI3K	phospho-phosphatidylinositide 3-kinases	DPD	Dihydropyrimidine dehydrogenase
p-AKT	phospho-american karate tae	TRAF6	TNF receptor-associated factor 6
GSK3*β*	Glycogen synthase kinase-3*β*	NF-κB	Nuclear Factor-κB
GOT	Glutamic oxalate transaminase	MCV	Mean corpuscular volume
GPT	Glutamic pyruvate transaminase	RDW	Red blood cell distribution width
NOAEL	No-observed-adverse-effect level	TGF-*β*1	Transforming Growth Factor *β*1
WBC	White blood cell	IL-1	Interleukin 1
HCT	Hematocrit	AST	Aspartate aminotransferase
RBC	Red blood cell	ALT	Alanine aminotransferase
LDH	Lactate dehydrogenase	IFN-*γ*	Interferon-*γ*
LN	Laminin	CTX	Cyclophosphamide
GSH-Px	Glutathione peroxidase	IL-6	Interleukin-6
NOS	Nitric oxide synthase	NO	Nitric oxide
AMPK	Adenosine phosphate-activated protein kinase	PGE2	Prostaglandin E2
PGC1	Peroxisome proliferator-activated receptor γ coactivator-1	EGF	Epidermal growth factor
BUN	Blood urea nitrogen	PAF	Platelet-activating factor
Cr	Creatinine	MES	Maximum electroconvulsive seizure
MRSA	Methicillin-resistant staphylococcus aureus	LOAEL	Lowest-observed-adverse-effect level
