# Supplementary material for: Research Progress in Traditional Applications, Phytochemistry, Pharmacology, and Safety Evaluation of Cynomorium songaricum
Source: Molecules. 2024 Feb 21;29(5):941. doi: 10.3390/molecules29050941 (PMC10935076; doi:10.3390/molecules29050941)
Supplement: Supplementary file 1 [file molecules-29-00941-s001.zip › molecules-2850917-supplementary.pdf]

Table S1. *Cynomorium songaricum* anti-tumor effects.

Effects	Detail	Source	Extracts/Compounds	Concentration/Dose	Control concentration/Dose	In vivo/In vitro	References
Anti-cancer	inhibition of MDA-MB-231 cell proliferation	Stems	95% ethanol extract	IC_{50} = 30.22 μg/mL	Vinblastine (10 μg/mL)	In vitro	[15]
	inhibition of MDA-MB-231 cell proliferation	75% ethanol extract	ethyl acetate part	IC_{50} = 34.48 ± 7.99 μg/mL	-	In vitro	[66]
	inhibition of MCF-7 cell proliferation	Stems	95% ethanol extract	IC_{50} = 37.03 μg/mL	Vinblastine (10 μg/mL)	In vitro	[15]
	inhibition of MCF-7 cell proliferation	75% ethanol extract	ethyl acetate part	IC_{50} = 30.93 ± 2.95 μg/mL	-	In vitro	[66]
	inhibition of MB468 cell proliferation	Stems	95% ethanol extract	IC_{50}=46.58 μg/mL	Vinblastine (10 μg/mL)	In vitro	[15]
	inhibition of 4T1 cell proliferation	Stems	95% ethanol extract	IC_{50}=44.20 μg/mL	Vinblastine (10 μg/mL)	In vitro	[15]
	inhibition of 4T1 cell proliferation	Stems	aqueous extract	IC_{50}=35.71 μg/mL	Vinblastine (10 μg/mL)	In vitro	[15]
	inhibition of Caco-2 cell proliferation	95% ethanol extract	chloroform part	IC_{50}=88.5 μg/mL	-	In vitro	[7]
	inhibition of Caco-2 cell proliferation	95% ethanol extract	ethyl acetate part	IC_{50}=179.3 μg/mL	-	In vitro	[7]
	inhibition of HeLa cell proliferation	Stems	CSP	800 μg/mL	-	In vitro	[69]
	inhibition of KBWT cell proliferation	-	methanol extract	CC_{50}=50.85 ± 1.85 μg/mL	Vinblastine (CC_{50}= 0.004 ± 0.002 μM)	In vitro	[67]
	inhibition of KBWT cell proliferation	-	cyanidin 3-*O*-glucoside	CC_{50}=45.09 ± 2.35 μg/mL	Vinblastine (CC_{50}= 0.004 ± 0.002 μM)	In vitro	[67]
	inhibition of TERT mRNA expression in A549 cells promotes apoptosis	Stems	CSP	6 mL/L	-	In vitro	[70]
	inhibition of	Stems	95% ethanol	IC_{50}=48.53	Vinblastine (10 μg/mL)	In vitro	[15]

	B16 cell proliferation		extract	μg/mL			
	inhibition of B16 cell proliferation	Stems	aqueous extract	IC_{50}=33.86 μg/mL	Vinblastine (10 μg/mL)	In vitro	[15]
	inhibition of LNCaP cell proliferation	75% ethanol extract	ethyl acetate part	IC_{50}=27.54±3.98 μg/mL	-	In vitro	[66]
	inhibition of HepG2 cell proliferation	75% ethanol extract	ethyl acetate part	IC_{50}=18.55±0.73 μg/mL	-	In vitro	[66]
Leukaemi a	regulation of caspase-3 activity by mitochondrial pathway induces apoptosis of HL-60 cells	Stems	70% ethanol extract	300 μg/mL	-	In vitro	[68]
	inhibition of CCRF-CEM cell proliferation		methanol extract	CC_{50}=12.15±0.0 07 μg/mL	Vinblastine (CC_{50}= 0.00277 ± 0.00055 μM)	In vitro	[67]
	inhibition of CCRF-CEM cell proliferation		cyanidin 3-*O*-glucoside	CC_{50}=1.5 ± 0.008 μg/mL	Vinblastine (CC_{50}= 0.00277 ± 0.00055 μM)	In vitro	[67]
	inhibition of CCRF-SB cell proliferation		methanol extract	CC_{50}=17.18±1.0 1 μg/mL	Vinblastine (CC_{50}= 0.002 μM)	In vitro	[67]
	inhibition of CCRF-SB cell proliferation		cyanidin 3-*O*-glucoside	CC_{50}=13.46±0.8 5 μg/mL	Vinblastine (CC_{50}= 0.002 μM)	In vitro	[67]

Table S2. *Cynomorium songaricum* anti-oxidation function.

Effects	Detail	Source	Extracts/Compounds	Concentration/Dose	Control concentration/Dose	In vivo/In vitro	References
Anti-oxidation function	scavenging O_2^-	Stems	ethyl acetate part	IC_{50}=2.9±0.3 μg/mL	Quercetin (IC_{50}=10.5±1.1 μg/mL)	In vitro	[73]
	scavenging O_2^-	Stems	methanol extract	IC_{50}=21.2±3.1 μg/mL	Quercetin (IC_{50}=10.5±1.1 μg/mL)	In vitro	[73]
	scavenging O_2^-	Stems	CSP	0.2 mg/mL, 0.4 mg/mL, 0.6 mg/mL、0.8 mg/mL, 1.0 mg/mL	VC (0.2 mg/mL, 0.4 mg/mL, 0.6 mg/mL, 0.8 mg/mL, 1.0 mg/mL)	In vitro	[6]
	scavenging O_2^-	Upper, middle	50% methanol extract	50 mg/mL	VC (1 mg/mL)	In vitro	[71]

	and lower parts					
scavenging O_2^-	Stems	aqueous extract	IC_{50}=1.01 mg/mL	VC (10–30 μg/mL)	In vitro	[74]
scavenging DPPH	Stems	80% ethanol extract	EC_{50}=53.72±0.46 μg/mL	VC (EC_{50}=34.97±0.94 μg/mL)	In vitro	[75]
scavenging DPPH	Stems	aqueous extract	EC_{50}=49.46±0.37 μg/mL	VC (EC_{50}=34.97±0.94 μg/mL)	In vitro	[75]
scavenging DPPH	Stems	70% acetone extract	IC_{50}=60.3±5.5 μg/mL	-	In vitro	[78]
scavenging DPPH	aqueous extract	N-hexane part	IC_{50}=861.2±130.7 μg/mL	-	In vitro	[78]
scavenging DPPH	aqueous extract	chloroform part	IC_{50}=350.3±151.0 μg/mL	-	In vitro	[78]
scavenging DPPH	aqueous extract	ethyl acetate part	IC_{50}=47.7±15.4 μg/mL	-	In vitro	[78]
scavenging DPPH	aqueous extract	N-butanol part	IC_{50}=45.8±7.5 μg/ mL	-	In vitro	[78]
scavenging DPPH	ethyl acetate part	protocatechuic acid	IC_{50}=25.0±13.8 μg/mL	VC (IC_{50}=10.7±0.1 μg/mL), VE (IC_{50}=23.4±0.8 μg/mL)	In vitro	[78]
scavenging DPPH	ethyl acetate part	gallic acid	IC_{50}=8.0±1.2 μg/mL	VC (IC_{50}=10.7±0.1 μg/mL), VE (IC_{50}=23.4±0.8 μg/mL)	In vitro	[78]
scavenging DPPH	ethyl acetate part	catechin	IC_{50}=10.2±2.3 μg/mL	VC (IC_{50}=10.7±0.1 μg/mL), VE (IC_{50}=23.4±0.8 μg/mL)	In vitro	[78]
scavenging DPPH	aqueous extract	catechin	IC_{50}=16.97±0.02 μg/mL	Rutin (IC_{50}=21.49±0.15 μg/mL)	In vitro	[79]
scavenging DPPH	aqueous extract	epicatechin	IC_{50}=11.15±0.09 μg/mL	Rutin (IC_{50}=21.49±0.15 μg/mL)	In vitro	[79]
scavenging DPPH	aqueous extract	oleuropein	IC_{50}=41.32±0.20 μg/mL	Rutin (IC_{50}=21.49±0.15 μg/mL)	In vitro	[79]
scavenging DPPH	95% ethanol extract	ethyl acetate part	31.25 μg/mL, 62.5 μg/mL, 125 μg/mL、250 μg/mL, 500 μg/mL	VC (31.25 μg/mL, 62.5 μg/mL, 125 μg/mL, 250 μg/mL, 500 μg/mL)	In vitro	[4]
scavenging DPPH	Fresh stems	aqueous extract	62.5 μg/mL, 125 μg/mL, 250 μg/mL, 500 μg/mL	VC (31.25 μg/mL, 62.5 μg/mL, 125 μg/mL, 250 μg/mL, 500 μg/mL)	In vitro	[4]
scavenging	Stems	CSP	0.2 mg/mL, 0.4	VC (0.2 mg/mL, 0.4	In vitro	[6]

DPPH			mg/mL, 0.6 mg/mL, 0.8 mg/mL, 1.0 mg/mL	mg/mL, 0.6 mg/mL, 0.8 mg/mL, 1.0 mg/mL)		
scavenging DPPH	80% ethanol extract	CSF	IC_{50}=0.469 mg/mL	VC (0.1 mg/mL, 0.2 mg/mL, 0.3 mg/mL, 0.4 mg/mL, 0.5 mg/mL, 0.6 mg/mL)	In vitro	[76]
scavenging DPPH	Stems	procyanidin	IC_{50}=0.224 mg/mL	VC (-)	In vitro	[39]
scavenging DPPH	Stems	crude polysaccharide	IC_{50}=145.6 μg/mL	VC (-), VE (-)	In vitro	[77]
scavenging DPPH	Stems	crude polyphenol	IC_{50}=79.3 μg/mL	VC (-), VE (-)	In vitro	[77]
scavenging DPPH	Upper, middle and lower parts	50% methanol extract	50 mg/mL	VC (1 mg/mL)	In vitro	[71]
scavenging DPPH	Stems	aqueous extract	IC_{50}=0.45 mg/mL	VC (1–10 μg/mL)	In vitro	[74]
scavenging ABTS	Stems	70% acetone extract	2.80±0.06 mM	-	In vitro	[78]
scavenging ABTS	aqueous extract	N-hexane part	0.042±0.002 mM	-	In vitro	[78]
scavenging ABTS	aqueous extract	chloroform part	0.36±0.07 mM	-	In vitro	[78]
scavenging ABTS	aqueous extract	ethyl acetate part	4.27±0.42 mM	-	In vitro	[78]
scavenging ABTS	aqueous extract	N-butanol part	1.79±0.08 mM	-	In vitro	[78]
scavenging ABTS	ethyl acetate part	protocatechuic acid	5.93±0.67 mM	VC (4.65±0.33 mM)、VE (1.67±0.02 mM)	In vitro	[78]
scavenging ABTS	ethyl acetate part	gallic acid	8.06±0.96 mM	VC (4.65±0.33 mM)、VE (1.67±0.02 mM)	In vitro	[78]
scavenging ABTS	ethyl acetate part	catechin	8.25±0.09 mM	VC (4.65±0.33 mM)、VE (1.67±0.02 mM)	In vitro	[78]
scavenging ABTS	95% ethanol extract	ethyl acetate part	250 μg/mL, 500 μg/mL	VC (31.25 μg/mL, 62.5 μg/mL, 125 μg/mL, 250 μg/mL, 500 μg/mL)	In vitro	[4]
scavenging ABTS	Fresh stems	aqueous extract	31.25 μg/mL, 62.5 μg/mL	VC (31.25 μg/mL, 62.5 μg/mL, 125 μg/mL, 250 μg/mL, 500 μg/mL)	In vitro	[4]

scavenging ABTS	Stems	crude polysaccharide	TEAC=0.69 mmol/g	VC (-), VE (-)	In vitro	[77]
scavenging ABTS	Stems	crude polyphenol	TEAC=2.12 mmol/g	VC (-), VE (-)	In vitro	[77]
scavenging OH^-	95% ethanol extract	ethyl acetate part	31.25 μg/mL, 62.5 μg/mL	VC (31.25 μg/mL, 62.5 μg/mL, 125 μg/mL, 250 μg/mL, 500 μg/mL)	In vitro	[4]
scavenging OH^-	Fresh stems	aqueous extract	31.25 μg/mL, 62.5 μg/mL, 250 μg/mL, 500 μg/mL	VC (31.25 μg/mL, 62.5 μg/mL, 125 μg/mL, 250 μg/mL, 500 μg/mL)	In vitro	[4]
scavenging OH^-	Stems	CSP	0.2 mg/mL, 0.4 mg/mL, 0.6 mg/mL, 0.8 mg/mL, 1.0 mg/mL	VC (0.2 mg/mL, 0.4 mg/mL, 0.6 mg/mL, 0.8 mg/mL, 1.0 mg/mL)	In vitro	[6]
scavenging OH^-	80% ethanol extract	CSF	IC_{50}=0.445 mg/mL	VC (0.1 mg/mL, 0.2 mg/mL, 0.3 mg/mL, 0.4 mg/mL, 0.5 mg/mL, 0.6 mg/mL)	In vitro	[76]
scavenging OH^-	Stems	procyanidin	IC_{50}=0.236 mg/mL	VC (-)	In vitro	[39]
scavenging OH^-	Upper, middle and lower parts	50% methanol extract	50 mg/mL	VC (1 mg/mL)	In vitro	[71]
total reducing ability	Stems	CSP	0.2 mg/mL, 0.4 mg/mL, 0.6 mg/mL, 0.8 mg/mL, 1.0 mg/mL	VC (0.2 mg/mL, 0.4 mg/mL, 0.6 mg/mL, 0.8 mg/mL, 1.0 mg/mL)	In vitro	[6]
scavenging NIT	Stems	80% ethanol extract	EC_{50}=1128.31±7.01 μg/mL	VC (EC_{50}=152.87±0.06 μg/mL)	In vitro	[75]
scavenging NIT	Stems	aqueous extract	EC_{50}=653.15±11.03 μg/mL	VC (EC_{50}=152.87±0.06 μg/mL)	In vitro	[75]
inhibition of SOD	Stems	80% ethanol extract	EC_{50}=951.70±2.45 μg/mL	VC (EC_{50}=41.83±1.00 μg/mL)	In vitro	[75]
inhibition of SOD	Stems	aqueous extract	EC_{50}=1787.03±14.35 μg/mL	VC (EC_{50}=41.83±1.00 μg/mL)	In vitro	[75]
scavenging XO/XTT	Stems	80% ethanol extract	EC_{50}=112.47±0.11 μg/mL	VC (EC_{50}=192.50±0.32 μg/mL)	In vitro	[75]

scavenging XO/XTT	Stems	aqueous extract	EC_{50}=561.19±26.99 μg/mL	VC (EC_{50}=192.50±0.32 μg/mL)	In vitro	[75]
scavenging XO/XTT	Stems	methanol extract	1 μg/mL, 10 μg/mL, 100 μg/mL	Quercetin (1 μg/mL, 10 μg/mL, 100 μg/mL)	In vitro	[72]
scavenging XO/XTT	methanol extract	dichloromethane part	1 μg/mL, 10 μg/mL, 100 μg/mL	Quercetin (1 μg/mL, 10 μg/mL, 100 μg/mL)	In vitro	[72]
scavenging XO/XTT	methanol extract	ethyl acetate part	1 μg/mL, 10 μg/mL, 100 μg/mL	Quercetin (1 μg/mL, 10 μg/mL, 100 μg/mL)	In vitro	[72]
scavenging XO/XTT	methanol extract	aqueous part	1 μg/mL, 10 μg/mL, 100 μg/mL	Quercetin (1 μg/mL, 10 μg/mL, 100 μg/mL)	In vitro	[72]
the DPPH scavenging activity in serum of KM mice was increased	Stems	60% ethanol extract	0.22 g/kg	-	In vivo	[80]

Table S3. *Cynomorium songaricum* anti-aging effects.

Effects	Detail	Source	Extracts/Compounds	Concentration/Dose	Control concentration/Dose	In vivo/In vitro	References
Anti-aging effects	inhibit the shortening of telomere length in blood cells and brain cells of aging model KM mice	Stems	CSP	20 mg/kg, 40 mg/kg, 80 mg/kg	APS (40 mg/g)	In vivo	[83]
	improve the aging model of male KM mice germ cell telomerase activity	Stems	CSP	20 mg/kg, 40 mg/kg, 80 mg/kg	-	In vivo	[84]
	improve immune function of aging model KM mice	Stems	CSP	20 mg/kg, 40 mg/kg, 80 g/kg	-	In vivo	[84]
	improve immune function of aging model Wistar rat	Stems	aqueous extract	1 g/kg	VE (0.025 g/kg)	In vivo	[85]

improve immune function of aging model Wistar rat	Stems	CSP	0.25 g/kg	VE (0.025 g/kg)	In vivo	[85]
improve immune function of aging model Wistar rat	Stems	CSP	0.25 g/kg	VE (0.025 g/kg)	In vivo	[86]
improve immune function of aging model Wistar rat	Stems	aqueous extract	1 g/kg	VE (0.025 g/kg)	In vivo	[87]
improve immune function of aging model Wistar rat	Stems	CSP	0.25 g/kg	VE (0.025 g/kg)	In vivo	[87]
inhibition of neuronal apoptosis in aging male KM mice	Stems	aqueous extract	2.6 g/kg	-	In vivo	[17]
inhibition of neuronal apoptosis in aging male KM mice	Stems	nano suspension	0. 5 g/kg	-	In vivo	[17]
inhibition of neuronal apoptosis in aging male KM mice	Stems	nano suspension	0. 5 g/kg	-	In vivo	[17]
improve hippocampal CA1 neurons in aging male KM mice	aqueous extract piece	ethyl acetate extract	0.02 g/kg	VE (4 mg/kg), Donepezil (0.71 mg/kg)	In vivo	[88]
improve hippocampal CA1 neurons in aging male KM mice	aqueous extract piece	95% ethanol extract	0.04 g/kg	VE (4 mg/kg), Donepezil (0.71 mg/kg)	In vivo	[88]
improve hippocampal CA1 neurons in aging male KM mice	aqueous extract piece	aqueous extract	0.17 g/kg	VE (4 mg/kg), Donepezil (0.71 mg/kg)	In vivo	[88]
alleviates the	Stems	aqueous extract	1.0 g/kg	-	In vivo	[90]

	damage of free radicals to mitochondrial membrane structure and function in aging male KM mice						
	increased scavenging of free radicals in KM mice	Stems	CSP	100 mg/kg, 200 mg/kg, 400 mg/kg, 800 mg/kg	-	In vivo	[89]
	reduced lipid peroxidation in KM female mice	Stems	CSF	0.08 g/kg, 0.17 g/kg, 0.50 g/kg	-	In vivo	[40]
	the extension of the lifespan of *C. elegans*	aqueous extract piece	ethyl acetate extract	0.4 mg/mL	-	In vivo	[82]

Table S4. *Cynomorium songaricum* anti-fatigue and anti-hypoxia activities.

Effects	Detail	Source	Extracts/Compounds	Concentration/Dose	Control concentration/Dose	In vivo/In vitro	References
Anti-fatigue	the ratio of cAMP/cGMP in KM male mice was decreased	Stems	aqueous extract	2 g/kg	TSPG (0.2 g/kg)	In vivo	[18]
	improve energy metabolism in KM mice	Stems	75% ethanol extract	0.125 g/mL, 0.25 g/mL, 0.5 g/mL	Rhodiola oral liquid (7 mL/kg), VE (5 mL/kg)	In vivo	[94]
	the exercise endurance, oxidative stress ability and energy metabolism of KM mice were enhanced	Stems	75% ethanol extract	0.125 g/mL、0.25 g/mL, 0.5 g/mL	Rhodiola oral liquid (7 mL/kg), VE (5 mL/kg)	In vivo	[94]
	enhance the activity of antioxidant enzymes in skeletal muscle of KM male mice	Stems	aqueous extract	1.0 g/mL	-	In vivo	[91]

	increase the hemoglobin of KM mice blood	Stems	aqueous extract	0.1 g/kg	-	In vivo	[100]
	the blood lactic acid index of KM male mice was decreased	Stems	aqueous extract	10 g/kg	-	In vivo	[91]
	inhibition of MAO in aged male Wistar rats	-	CSF	0.5 g/kg, 1.0 g/kg, 2.0 g/kg	-	In vivo	[97]
	increase the swimming time of Wistar male rats	-	CSF	0.5 g/kg, 1.0 g/kg, 2.0 g/kg	-	In vivo	[98]
	improve free radical metabolism in skeletal muscle of male Wister rats	Stems	aqueous extract	0.8 mL/kg	-	In vivo	[95]
	improve free radical metabolism in Wistar male rats, inhibit ROS	Stems	CSF	0.5 g/kg, 1.0 g/kg, 2.0 g/kg	-	In vivo	[96]
	increase exercise Wistar male rat muscle glycogen	Stems	aqueous extract	3.5 g/kg	-	In vivo	[92]
	inhibit the decomposition of amino acids and proteins and increase the reserve of hemoglobin and glycogen in male Wister rats.	Stems	aqueous extract	0.75 g/kg, 1.5 g/kg, 4.5 g/kg	-	In vivo	[93]
Anti-hypoxia	increase the hemoglobin of KM mice blood	Stems	aqueous extract	0.1 g/kg	-	In vivo	[100]

the survival time and opening times of KM mice were improved	Stems	aqueous extract	1.0 g/mL	-	In vivo	[99]
increased myocardial protein in male BALB/C mice	Stems	aqueous extract partIII	300 mg/kg	TSPG (300 mg/kg)	In vivo	[101]

Table S5. *Cynomorium songaricum* effects on nervous system.

Effects	Detail	Source	Extracts/Compounds	Concentration/ Dose	Control concentration/Dose	In vivo/In vitro	References
Effects on nervous system	protect SK-N-SH cells from Aβ_{25-35}-induced injury	overground part	methanol extract	100 μg/mL, 10 μg/mL	-	In vitro	[102]
	protect SK-N-SH cells from Aβ_{25-35}-induced injury	overground part	ethyl acetate extract	100 μg/mL, 10 μg/mL	-	In vitro	[102]
	protect SK-N-SH cells from HPX / XO-induced damage	overground part	methanol extract	1.0 μg/mL	-	In vitro	[102]
	protect SK-N-SH cells from HPX / XO-induced damage	overground part	ethyl acetate extract	1.0 μg/mL, 0.1 μg/mL	-	In vitro	[102]
	protect SK-N-SH cells from XDH / XO-induced damage	Stems	methanol extract	1 μg/mL, 10 μg/mL, 100 μg/mL	-	In vitro	[72]
	protect SK-N-SH cells from XDH / XO-induced damage	Stems	ethyl acetate extract	1 μg/mL, 10 μg/mL, 100 μg/mL	-	In vitro	[72]
	protect SK-N-SH cells from staurosporine-induced damage	Stems	ethyl acetate extract	100 μg/mL, 10 μg/mL	-	In vitro	[73]
	protect PC12 cells from H_2O_2-	Stems	CSP	0.5 mg/mL, 1.0 mg/mL	-	In vitro	[103]

induced injury						
protect PC12 cells from Aβ_{25-35}-induced injury	95% ethanol extract	ethyl acetate part	0.83 mg/kg, 8.3 mg/kg, 83 mg/kg	-	In vitro	[104]
reduce the phosphorylation of Drp1 at Ser637 in Aβ_{25-35}-induced HT22 cell model	aqueous extract piece	methanol extract	200 μg/mL	-	In vitro	[106]
reduce the expression of Fis1 in H_2O_2-induced HT22 cell model	aqueous extract piece	methanol extract	200 μg/mL	-	In vitro	[106]
inhibition of Neuro2A cell	Stems	ethyl acetate extract	EC_{50}=116 mg/L	-	In vitro	[105]
up-regulation of synaptophysin expression via MAPK pathway	Stems	ethyl acetate extract	100 mg/L	-	In vitro	[105]
enhance hippocampal plasticity in male C57BL / 6J mice	Stems	80% ethanol extract	40 mg/kg, 100 mg/kg	-	In vivo	[114]
up-regulation of BDNF / TrkB signaling pathway in hippocampus of Wistar male rats	-	CSF	25 mg/kg, 50 mg/kg, 100 mg/kg	Donepezil (0.5 mg/kg)	In vivo	[116]
the expression of NADPH oxidase, ROS and NLRP3 in hippocampus of male Wistar rats was down-regulated	65% ethanol extract	CSF	25 mg/kg, 50 mg/kg, 100 mg/kg	Donepezil (0.5 mg/kg)	In vivo	[117]
protect SD rat hippocampal neurons from kainic acid-induced injury	Stems	ursolic acid	5–10 μM	-	In vivo	[118]
inhibition of p38MAPK / ERK pathway increases CREB / BDNF	Stems	95% ethanol extract	3.3 mg/kg, 33 mg/kg	estradiol valerate (2 mg/kg)	In vivo	[113]

expression in ovariectomized SD rats						
increase the GAP-43 protein in the hippocampus of ovariectomized SD rats	70% ethanol extract	ethyl acetate part	33 mg/kg	estradiol valerate (2 mg/kg)	In vivo	[109]
the expression of p-CREB in ovariectomized SD rats was increased and the expression of p38 was decreased by MAPK pathway	70% ethanol extract	ethyl acetate part	3.3 mg/kg	estradiol valerate (2 mg/kg)	In vivo	[119]
up-regulation of P-Erk1 / 2 and P-CREB in MAPK signaling pathway	70% ethanol extract	ethyl acetate part	0.47 mg/mL	-	In vivo	[111]
improve cognitive dysfunction in chronic stress ICR mice after ovariectomy	70% ethanol extract	ethyl acetate part	0.47 mg/mL	-	In vivo	[112]
increase the expression of Syn and PSD-95 in ovariectomized ICR mice	70% ethanol extract	ethyl acetate part	0.47 mg/mL	-	In vivo	[110]
reduce oxidative stress in brain tissue of scopolamine KM male mice	Stems	aqueous extract	5 g/kg, 10 g/kg	Piracetam (100 mg/kg)	In vivo	[115]
improve the expression of OPA1, MFN1 and DRP1 protein in AD model C57BL / 6 male mice	70% ethanol extract	ethyl acetate part	47 mg/kg	-	In vivo	[107]
improve the intestinal flora disorder of AD	70% ethanol extract	ethyl acetate part	47 mg/kg	-	In vivo	[108]

	model C57BL / 6 male mice						

Table S6. *Cynomorium songaricum* effects on reproductive system.

Effects	Detail	Source	Extracts/Compounds	Concentration/ Dose	Control concentration/Dose	In vivo/In vitro	References
Effects on reproductive system	the expression of AR and ERα was down-regulated and the expression of ERβ was up-regulated in BPH-1 cells	-	Luteolin、Gallic acid、Ferulic acid、Protocatechualdehyde	-	-	In vitro	[123]
	inhibition of 5α-reductase	Stems	75% ethanol extract	0.02 mg/mL, 0.1 mg/mL, 0.5 mg/mL, 2.5 mg/mL	-	In vitro	[121]
	the expression of PCNA, AR and ERα protein in Wistar rats was inhibited, and the expression of ERβ protein was promoted	Stems	60% ethanol extract	3 g/kg	Flutamide (30 mg/kg)	In vivo	[122]
	interference the expression of AR, ERα / β and SRD5A1 / 2 in the prostate of Wistar rats	Stems	70% ethanol extract	1.33 g/kg, 2.66 g/kg	Flutamide (30 mg/kg)	In vivo	[123]
	improving oxidative stress in prostate tissue of SD rats	Stems	aqueous extract	40 mg/kg, 80 mg/kg, 120 mg/kg	Qianliekang (570 mg/kg)	In vivo	[124]
	interference of estrogen / androgen signal in Wistar rats	Stems	70% ethanol extract	6 g/kg	-	In vivo	[58]
	promoting spermatogenesis in immature Wistar rats by testosterone-like effect	Fresh stems	aqueous extract	0.47 g/kg	-	In vivo	[127]
	up-regulate the expression of GDNF in the	Stems	aqueous extract	1.0 g/kg	-	In vivo	[131]

	testis of Wistar rats and enhance spermatogenesis						
	up-regulation of GDNF expression in the testis of SD rats	Stems	aqueous extract	0.5 g/kg, 1.0 g/kg, 2.0 g/kg	VE (50 mg/kg)	In vivo	[130]
	up-regulation of GDNF expression in the testis of SD rats	Stems	aqueous extract	0.5 g/kg, 1.0 g/kg, 2.0 g/kg	-	In vivo	[129]
	inhibition of abnormal secretion of FSH and LH in SD rats	Stems	aqueous extract	0.5 g/kg	-	In vivo	[128]
	promoting the spermatogenic ability of golden hamster testis degeneration	Stems	aqueous extract	0.5 g/kg, 1.5 g/kg, 2.5 g/kg	-	In vivo	[132]

Table S7. *Cynomorium songaricum* anti-virus.

Effects	Detail	Source	Extracts/Compounds	Concentration/ Dose	Control concentration/Dose	In vivo/In vitro	References
Anti-HCV	inhibit the HCV protease	dichloromethane extract	Ursolic acid	IC_{50}=16 μg/mL	Embelin (IC_{50}=21 μM)	In vitro	[47]
	inhibit the HCV protease	dichloromethane extract	Acetyl ursolic acid	IC_{50}=11 μg/mL	Embelin (IC_{50}=21 μM)	In vitro	[47]
	inhibit the HCV protease	dichloromethane extract	Malonyl ursolic acid hemiester	IC_{50}=3 μg/mL	Embelin (IC_{50}=21 μM)	In vitro	[47]
Anti-HIV	inhibit the HIV-1 protease	dichloromethane extract	Ursolic acid	IC_{50}=8 μM	Histone deacetylase inhibitor (IC_{50}=0.09 μM)	In vitro	[16]
	inhibit the HIV-1 protease	dichloromethane extract	Acetyl ursolic acid	IC_{50}=13 μM	Histone deacetylase inhibitor (IC_{50}=0.09 μM)	In vitro	[16]
	inhibit the HIV-1	dichloromethane extract	Malonyl ursolic acid hemiester	IC_{50}=6 μM	Histone deacetylase inhibitor (IC_{50}=0.09 μM)	In vitro	[16]

protease						
inhibit the HIV-1 protease	dichloromethane extract	3′,3′-dimethylglutaryl hemiester	IC_{50}=4 μM	Histone deacetylase inhibitor (IC_{50}=0.09 μM)	In vitro	[16]
inhibit the HIV-1 protease	dichloromethane extract	Benzylthioepicatechin	IC_{50}=2 μg/mL	Histone deacetylase inhibitor (IC_{50}=0.09 μM)	In vitro	[16]
inhibit the HIV-1 protease	dichloromethane extract	Malonyl oleanolic acid hemiester	IC_{50}=8 μM	Histone deacetylase inhibitor (IC_{50}=0.09 μM)	In vitro	[10]
inhibit the HIV protease	Stems	SCSP-M	EC_{50}=0.30 μg/mL	Dextran sulfate (EC_{50}=0.35 μg/mL), Curdlan sulfate (EC_{50}=0.14 μg/mL)	In vitro	[134]
inhibit the HIV protease	Stems	SCSP-1	EC_{50}=0.40 μg/mL	Dextran sulfate (EC_{50}=0.35 μg/mL), Curdlan sulfate (EC_{50}=0.14 μg/mL)	In vitro	[134]
inhibit the HIV protease	Stems	SCSP-2	EC50=0.30 μg/mL	Dextran sulfate (EC_{50}=0.35 μg/mL), Curdlan sulfate (EC_{50}=0.14 μg/mL)	In vitro	[134]

Table S8. *Cynomorium songaricum* anti-diabetic properties.

Effects	Detail	Source	Extracts/Compounds	Concentration/ Dose	Control concentration/Dose	In vivo/In vitro	References
Anti-diabetic properties	inhibit the *α*-glucosidase activity	Stems	4*β*-(L-Cysteinyl)-epicatechin	IC_{50}=78 μg/mL	-	In vitro	[64]
	inhibit the *α*-glucosidase activity	Stems	*β*-(L-cysteinyl)-epicatechin 3-*O*-gallate	IC_{50}=74 μg/mL	-	In vitro	[64]
	inhibit the *α*-glucosidase activity	Stems	Flavan-3-ol oligomers	IC_{50}=30 μg/mL	-	In vitro	[140]
	inhibit the sucrase activity	Stems	4*β*-(L-Cysteinyl)-epicatechin	IC_{50}=80 μg/mL	-	In vitro	[64]
	inhibit the sucrase activity	Stems	*β*-(L-cysteinyl)-epicatechin 3-*O*-gallate	IC_{50}=58 μg/mL	-	In vitro	[64]
	inhibit the maltase activity	Stems	4*β*-(L-Cysteinyl)-epicatechin	IC_{50}=61 μg/mL	-	In vitro	[64]
	inhibit the maltase activity	Stems	*β*-(L-cysteinyl)-epicatechin 3-*O*-gallate	IC_{50}=49 μg/mL	-	In vitro	[64]

	Wistar rats decreased blood sugar and increased insulin and liver glycogen	Stems	CSPA	200 mg/kg	Glibenclamide (200 mg/kg)	In vivo	[53]
	regulates phospholipid metabolism in C57BL/6 male mice	Stems	CSP	424 mg/kg, 212 mg/kg, 106 mg/kg	Metformin (168 mg/kg)	In vivo	[137]
	the expression of AKT and eNOS was up-regulated and TNF-α was down-regulated in SD male rats	Stems	CSP	300 mg/kg	-	In vivo	[136]

Table S9. *Cynomorium songaricum* anti-osteoporosis effect.

Effects	Detail	Source	Extracts/Compounds	Concentration/Dose	Control concentration/Dose	In vivo/In vitro	References
Anti-osteoporosis effect	promote UMR106 cell proliferation	Stems	methanol extract	400 μg/mL	-	In vitro	[139]
	promote MC3T3-E1 cell proliferation	Stems	aqueous extract	0.225 g/mL, 0.45 g/mL, 0.8 g/mL	-	In vitro	[142]
	activate the PI3K/AKT/GSK3β/β-catenin pathway in MC3T3-E1 osteoblasts	Stems	CSP	100 μg/mL	-	In vitro	[140]
	inhibition of LPS-induced apoptosis of MC3T3-E1 osteoblasts (up-regulated Bax and caspase-3, down-regulated Bcl-2)	Stems	80% ethanol extract	25 μM, 50 μM	-	In vitro	[141]
	activation of PI3K/AKT, NF-κB signaling pathway in ovariectomized	Stems	80% ethanol extract	100 mg/kg, 300 mg/kg	estradiol valerate (1 mg/kg)	In vivo	[145]

SD rats activation of OPG/RANK/RANKL signaling pathway in ovariectosed SD rats	-	CSP	20 mg/kg, 40 mg/kg, 80 mg/kg	Nilestriol (1.5 mg/kg)	In vivo	[143]
increased ALP in ovariectomized SD rats; Reduce TRAP, Cathepsin K, and DPD	Stems	80% ethanol extract	100 mg/kg, 300 mg/kg	estradiol valerate (1 mg/kg)	In vivo	[144]

Table S10. *Cynomorium songaricum* liver protection.

Effects	Detail	Source	Extracts/Compounds	Concentration/ Dose	Control concentration/Dose	In vivo/In vitro	References
Liver protection	protect female SD rats from CCl4 hepatotoxicity through mitochondrial pathway	95% ethanol extract	HCY2	0.5 mg/kg, 1.0 mg/kg	-	In vivo	[152]
	protect female SD rats from CCl_4 hepatotoxicity through mitochondrial pathway	-	UA	0.35 mg/kg, 0.70 mg/kg	-	In vivo	[152]
	reduce the expression of TGF-β_1 in SD male rats induced by CCl_4	Stems	CSR extract	50 mg/kg, 100 mg/kg, 200 mg/kg	Colchicine (0.2 mg/kg)	In vivo	[148]
	increase WBC, HCT, RBC, MCV and RDW in blood cells of SD male rats induced by CCl_4	Stems	CSR extract	200 mg/kg	Colchicine (0.2 mg/kg)	In vivo	[149]
	improve CCl_4-induced KM	Stems	60% ethanol extract	0.15 g/kg, 0.3 g/kg, 0.6 g/kg	silybin capsules (0.2 g/kg)	In vivo	[151]

male mice						
antioxidant and free radical scavenging capacity						
reduce CCl_4-induced TGF-β_1, TNF-α, IL-1 in the liver of C57BL / 6J male mice	Stems	CSR extract	2.77 mg/kg, 27.7 mg/kg	Colchicine (0.1 mg/kg)	In vivo	[150]
reduce STZ-induced liver injury in Wistar rats GOT, GPT	Stems	CSPA	200 mg/kg, 150 mg/kg	Glibenclamide (200 mg/kg)	In vivo	[53]
alleviate free radical damage to liver cells of male Wister rats	Stems	aqueous extract	3.5 g/kg	-	In vivo	[92]
the activities of SOD and GSH-Px in T6 cells induced by formaldehy de were increased	Stems	CSF	50 μg/mL, 100 μg/mL	-	In vitro	[153]
protect T6 cells from H_2O_2-induced oxidative damage	Stems	CSF	25 μg/mL, 50 μg/mL, 100 μg/mL	-	In vitro	[154]

Table S11. *Cynomorium songaricum* other pharmacological effects.

Effects	Detail	Source	Extracts/Compounds	Concentration/Dose	Control concentration/Dose	In vivo/In vitro	References
Intestinal effects	defecation time of KM mice is shortened	Stems	aqueous extract	3.9 g/kg	-	In vivo	[43]
	inhibition of intestinal peristalsis in KM mice	Stems	CSP	14.28 mg/kg, 28.57 mg/kg, 57.14 mg/kg	-	In vivo	[155]
	amplitude of	Stems	aqueous extract	0.01 g/mL,	-	In vivo	[156]

	smooth muscle contraction in the jejunum of New Zealand Great white rabbits is inhibited			0.015 g/mL, 0.02 g/mL			
Mitigate obesity	weight gain and fat pad weight were significantly reduced in male ICR mice	95% ethanol extract	HCY2	112 mg/kg, 336 mg/kg	-	In vivo	[157]
	weight gain and fat pad weight were significantly reduced in female ICR mice	95% ethanol extract	HCY2	180 mg/kg, 360 mg/kg	-	In vivo	[157]
	regulation of AMPK / PGC1 pathway in male ICR mice	95% ethanol extract	HCY2	112 mg/kg, 336 mg/kg	Orlistat (20 mg/kg)	In vivo	[158]
Renal protective effects	protect female SD rats from gentamicin nephrotoxicity through mitochondrial pathway	95% ethanol extract	HCY2	0.5 mg/kg, 1.0 mg/kg	-	In vivo	[152]
	protect female SD rats from gentamicin nephrotoxicity through mitochondrial pathway	-	UA	0.5 mg/kg, 1.0 mg/kg	-	In vivo	[152]
	reduce STZ-induced renal injury in Wistar rats BUN, Cr	Stems	CSPA	200 mg/kg, 150 mg/kg	Glibenclamide (200 mg/kg)	In vivo	[53]
	inhibition of H_2O_2-induced apoptosis of VERO cells	Stems	CSP	0.25 mg/mL, 0.5 mg/mL, 1.0 mg/mL	-	In vitro	[159]
Immune system modulation	enhance the phagocytic ability of macrophages in male BALB / C	Stems	aqueous extract partIII	300 mg/kg	TSPG (300 mg/kg)	In vivo	[160]

	mice and increase antibodies in serum						
	the secretion of IL-2, IFN-γ and TNF-α in serum of KM mice was increased	Stems	aqueous extract	0.18 g/kg, 0.36 g/kg, 0.72 g/kg	Levamisole (30 mg/kg)	In vivo	[14]
	the secretion of IL-2, IFN-γ and TNF-α in serum of KM mice was increased	Stems	75% ethanol extract	0.1 g/kg, 0.2 g/kg, 0.4 g/kg	Levamisole (30 mg/kg)	In vivo	[14]
	promote RAW264.7 proliferation, NO, phagocytosis, IL-6 and TNF-α	Stems	CSP	25–400 μg/mL	-	In vitro	[161]
Anti-ulcer effect	improve the microcirculation and defense ability of gastric mucosa in Wister rats	Stems	CSP	100 mg/kg, 200 mg/kg, 400 mg/kg	ranitidine hydrochloride capsules (50 mg/kg)	In vivo	[162]
	the levels of SOD, PGE2, and EGF were elevated in the serum of Wister rats, while MDA and PAF concentrations were reduced	Stems	CSP	100 mg/kg, 200 mg/kg, 400 mg/kg	ranitidine hydrochloride capsules (50 mg/kg)	In vivo	[163]
Anti-depressant effect	the hypothalamic-pituitary-gonadal axis was regulated by increasing E2 in female SD rats	-	CSF（50.87%）	0.20 g/kg, 0.10 g/kg, 0.05 g/kg	Gengnianan capsule (0.45 mg/kg)	In vivo	[164]
	the lesions of uterus, thymus, spleen and hypothalamus of KM female mice were improved	-	CSF	400 mg/kg, 200 mg/kg, 100 mg/kg	Gengnianan capsule (675 mg/kg)	In vivo	[165]
Anti-	reduce the	Stems	aqueous extract	1.0 g/mL	-	In vivo	[100]

epileptic	number of convulsions in KM mice						
Anti-bacterial	inhibition MRSA	Fresh stems	Cynomoriitannin	MIC=64 μg/mL	-	In vitro	[41]
